# DNA Barcoding and Untargeted Metabolomics Reveal Phylogenetic and Chemotaxonomic Relationships Among 11 *Ferula* Species from Xinjiang, China

**DOI:** 10.3390/metabo16100712

**Published:** 2026-09-24

**Authors:** Shuak Halimubek, Pengyan Yan, Lei Chen, Jingjing Chen, Xiaoqing Zhang, Siyu Chen, Dongdong Wang, Haiyan Xu, Xuejia Zhang

**Affiliations:** 1 College of Traditional Chinese Medicine, Xinjiang Medical University, Urumqi 830017, China; xuah0529@163.com (S.H.); 17590823073@163.com (P.Y.); chenlei@bucm.edu.cn (L.C.); 17799160495@163.com (J.C.); 15699091569@163.com (X.Z.); atmzpl@163.com (S.C.); wang_tcm@126.com (D.W.); xhy66003@163.com (H.X.); 2 School of Chinese Materia Medica, Beijing University of Chinese Medicine, Beijing 102401, China; 3 Institute of Traditional Chinese Medicine, Xinjiang Medical University, Urumqi 830017, China; 4 Xinjiang Laboratory of Famous Prescription and Science of Formulas, Xinjiang Medical University, Urumqi 830017, China; 5 Key Laboratory for Research on Planting Standards of Authentic Chinese Medicinal Materials in Xinjiang, Xinjiang Medical University, Urumqi 830017, China

**Keywords:** *Ferula*, DNA barcoding, untargeted metabolomics, chemotaxonomy, metabolic profiling, phylogenetic relationships, specialized metabolites

## Abstract

**Background/Objectives**: Morphological similarity among *Ferula* species, particularly during the vegetative stage, complicates the reliable authentication of leaf materials. The correspondence between phylogenetic relationships and leaf chemotaxonomic patterns among wild and cultivated *Ferula* resources remains insufficiently characterized. This study addressed three questions in 11 *Ferula* species from Xinjiang, China: whether combined DNA barcodes support species authentication and broad phylogenetic grouping; whether leaf metabolic profiles reveal taxon-associated differentiation; and to what extent phylogenetic and metabolic groupings correspond. **Methods**: Four DNA barcode loci, ITS2, *trnH–psbA*, *rps11*, and *atpA–trnR*, were combined for molecular authentication and phylogenetic analysis. Ultrasound-assisted extraction conditions were optimized using response surface methodology to establish standardized sample preparation, followed by LC–MS-based untargeted metabolomic profiling of 33 leaf samples representing 11 species. Multivariate analyses were used to characterize taxon-associated metabolic patterns and chemotaxonomic relationships. **Results**: The combined DNA barcode dataset generally supported the authentication of most investigated samples and revealed four broad phylogenetic groups, although several closely related accessions showed limited resolution. Untargeted metabolomics resulted in the putative annotation of 1972 metabolites, predominantly terpenoids, flavonoids, phenolic acids, and coumarins. PCA revealed taxon-associated metabolic patterns, while HCA grouped the samples into three major metabolic clusters. Notably, *F. sinkiangensis*, *F. fukanensis*, *F. lehmannii*, *F. teterrima*, and *F. conocaula* occurred within the same broad phylogenetic group and the same metabolic group, indicating partial correspondence between molecular relationships and metabolic phenotype. **Conclusions**: DNA barcoding and untargeted metabolomics provided complementary genetic and chemical evidence for the authentication and comparative characterization of *Ferula* leaves. The observed partial correspondence between phylogenetic relationships and metabolic profiles provides an exploratory basis for understanding chemical differentiation among the investigated *Ferula* species.

## 1. Introduction

The genus *Ferula* (Apiaceae) comprises approximately 180–185 species of perennial herbs distributed widely across Europe and Asia [1,2]. Many *Ferula* species are recognized worldwide as important medicinal, aromatic, and food-related resources due to their diverse traditional applications [1,2,3]. Approximately 30 *Ferula* species have been documented in China, with Xinjiang representing a major center of diversity and distribution [1,4]. The utilization of *Ferula* species has traditionally centered on oleo-gum-resins exuded from the roots and rhizomes [2,5]. These resins have been widely used in several traditional medical systems, including traditional Chinese medicine, Iranian traditional medicine, Ayurveda, and Arabian medicine, and particularly for the treatment of digestive and respiratory disorders, inflammatory conditions, rheumatic disorders, and skin diseases [2,3,5,6,7,8,9,10,11]. In addition, *Ferula* resins have been used as spices in India, Iran, and Afghanistan, because of their strong garlic-like flavor [11]. Phytochemical investigations have revealed that *Ferula* species are rich in secondary metabolites, including sesquiterpenes, coumarins, sulfur-containing compounds, flavonoids, and phenolic acids [5,8,12]. Extracts and isolated constituents from *Ferula* species have been reported to exhibit anticancer, antimicrobial, anti-inflammatory, neuroprotective, antiparasitic, and immunomodulatory activities [2,3,5,6,7,8,9,10,11,12]. However, previous studies have predominantly focused on the medicinal resins of *Ferula*, while the genetic authentication and chemical diversity of edible leaf resources remain comparatively less explored. In Xinjiang, the fresh leaves of several *Ferula* species, such as *F. sinkiangensis*, *F. fukanensis*, *F. lehmannii*, and *F. teterrima*, have long been consumed as vegetables and are traditionally considered beneficial for digestive health [13,14]. With the expansion of cultivation and increasing utilization of *Ferula* leaves, accurate species authentication and characterization of their chemical diversity have become increasingly important.

However, the provenance and taxonomic identity of *Ferula* materials are not always clearly documented during cultivation, harvest, and commercial circulation, and leaves derived from different species, geographical origins, or wild and cultivated populations may become mixed. The leaves of closely related *Ferula* species often exhibit similar morphology, making it difficult to distinguish individual species [14,15]. Moreover, fresh leaves are commonly blanched and frozen for preservation when not consumed immediately, which can further reduce the diagnostic value of morphological characteristics. Such difficulties are particularly relevant because metabolite composition and accumulation patterns may vary among *Ferula* species, and species admixture may therefore obscure taxon-associated chemical profiles and compromise the consistency and chemical evaluation of *Ferula* leaf resources [14,16]. Consequently, reliable approaches capable of accurately authenticating morphologically similar *Ferula* leaves and characterizing their interspecific chemical differences are needed.

DNA barcoding has become a widely used approach for plant identification and authentication. By targeting standardized genetic loci, such as ITS2 and chloroplast regions, this method requires relatively small amounts of biological material, provides high reproducibility, and does not depend on the availability of complete morphological characteristics [17,18,19]. It is therefore particularly useful for identifying plant taxa with highly similar morphology [20]. However, the discriminatory power of conventional DNA barcodes may be reduced for closely related or recently diverged taxa and in cases involving hybridization or introgression [21,22]. Metabolomics provides complementary information by comprehensively characterizing metabolite composition and relative abundance, thereby enabling the assessment of interspecific chemical variation and chemotaxonomic relationships [16,23]. Although plant metabolic profiles are shaped by genetic background as well as environmental and developmental factors, comparative metabolomics can reveal taxon-associated metabolic patterns that complement molecular identification [24]. Accordingly, integrated approaches combining DNA barcoding, molecular phylogeny, and metabolomics have increasingly been applied to the authentication, chemotaxonomic evaluation, and chemical characterization of medicinal and edible plant resources [14,25,26,27].

Recent plastid genomic studies have improved our understanding of phylogenetic relationships within *Ferula* and related taxa and have identified highly variable plastid regions with potential value for species identification [15]. These studies demonstrate the usefulness of genome-scale molecular information for resolving taxonomic relationships; however, conventional multi-locus DNA barcoding remains a practical strategy for routine authentication when rapid and targeted species identification is required. In parallel, metabolomics has been increasingly applied to discriminate *Ferula* species and to characterize interspecific variation in their chemical profiles [14,16]. Nevertheless, most previous studies have focused on a limited number of species, particular cultivated populations, or either molecular or metabolomic evidence alone. Comparative studies integrating DNA-based phylogenetic relationships with leaf metabolic profiles across a broader range of wild and cultivated *Ferula* resources from Xinjiang remain limited. Such an integrated comparison is needed to determine the extent to which phylogenetic relatedness is reflected in chemotaxonomic and metabolic patterns among *Ferula* species.

To address these gaps, 33 leaf samples representing 11 *Ferula* species from diverse wild and cultivated resources in Xinjiang, China, were investigated using an integrated strategy combining multi-locus DNA barcoding and LC–MS-based untargeted metabolomics. DNA barcodes were used for molecular authentication and phylogenetic reconstruction, while ultrasound-assisted extraction conditions were optimized to establish standardized sample preparation for subsequent metabolomic profiling. The metabolomic data were then used to characterize interspecific chemical variation, identify metabolites contributing to group differentiation, and evaluate chemotaxonomic relationships among the investigated taxa. Particular emphasis was placed on assessing the extent to which DNA-based phylogenetic relationships corresponded to metabolomic grouping patterns across this broader taxonomic range, as well as on examining the metabolic characteristics of species with documented culinary use. By integrating molecular and chemical evidence, this study aimed to provide a comparative framework for the authentication of morphologically similar *Ferula* leaves and for understanding the relationships among phylogenetic affinity, metabolic diversity, and chemotaxonomic differentiation in *Ferula* resources from Xinjiang.

## 2. Materials and Methods

### 2.1. Plant Materials

Between 2023 and 2025, leaf samples of different *Ferula* species were collected from multiple locations in Xinjiang, China, representing both wild populations and cultivated resources. All plant materials were authenticated by Haiyan Xu and Xuejia Zhang from Xinjiang Medical University based on diagnostic morphological characteristics, including reproductive traits observed during the flowering and fruiting stages. Voucher specimens were deposited in the Herbarium of Xinjiang Medical University. Detailed information on species identity, collection location and date, wild or cultivated status, and voucher numbers is provided in Table S1. The collected leaf materials were used for DNA barcoding and LC–MS-based untargeted metabolomic analysis. For DNA barcoding, fresh and healthy leaves were individually placed in labeled resealable bags immediately after collection and rapidly dried with silica gel to minimize DNA degradation and microbial contamination. For metabolomic analysis, leaf samples were shade-dried under cool and well-ventilated conditions, ground into a fine powder, transferred to sealed containers, and stored until extraction and subsequent LC–MS analysis.

### 2.2. DNA Extraction and PCR Amplification

Approximately 20 mg of dried leaf material was ground into a fine powder under liquid nitrogen. Total genomic DNA was extracted using a genomic DNA extraction kit (Tiangen Biotech, Beijing, China) according to the manufacturer’s instructions. PCR amplification was performed using a TC-96/G/H(b)B thermal cycler (Hangzhou Bioer Technology Co., Ltd., Hangzhou, China) in a final reaction volume of 25 μL containing 2 μL of template DNA (20–30 ng/μL), 1 μL each of forward and reverse primers (Table 1), 12.5 μL of 2× Taq PCR MasterMix, and 8.5 μL of double-distilled water. The amplification program consisted of an initial denaturation at 94 °C for 5 min, followed by 30 cycles of denaturation at 94 °C for 30 s, annealing at 55 °C for 30 s, and extension at 72 °C for 45 s, with a final extension at 72 °C for 10 min. The PCR products were examined by electrophoresis on a 1.5% agarose gel, and products showing single and distinct bands were selected for bidirectional sequencing by Sangon Biotech (Shanghai, China). The PCR amplification procedures were performed based on previously established plant DNA barcoding protocols with minor modifications [19,28,29,30,31].

### 2.3. Phylogenetic Tree Construction

Following bidirectional sequencing, the forward and reverse reads were manually inspected and edited. Low-quality bases at both ends and regions containing ambiguous chromatogram peaks were removed, and the corresponding forward and reverse reads were assembled into consensus sequences. Sequences from the four barcode loci (ITS2, *trnH–psbA*, *rps11*, and *atpA–trnR*) were aligned separately and subsequently concatenated to generate a combined DNA barcode dataset. The alignments were manually inspected and refined, and poorly aligned regions and terminal positions with inconsistent sequence coverage were excluded. Only confidently aligned homologous sites were retained for subsequent phylogenetic analyses. Three Apiaceae taxa with adequate sequence coverage across the concatenated loci, *Angelica sinensis*, *Bupleurum commelynoideum*, and *Chamaesium jiulongense*, were selected as outgroups for the final phylogenetic analyses (Table S2).

Phylogenetic analyses were conducted in MEGA 12. The Neighbor-Joining (NJ) tree was reconstructed using the Maximum Composite Likelihood distance method, with gaps and missing data treated by pairwise deletion. Branch support was evaluated using 1000 bootstrap replicates.

To further evaluate the robustness of the NJ-based relationships, an additional Maximum Likelihood (ML) analysis was performed using the same taxon set. The best-fitting nucleotide substitution model was selected according to the Bayesian Information Criterion (BIC), and the Tamura 3-parameter model with gamma-distributed rates and a proportion of invariant sites (T92+G+I) was selected as the optimal model. The ML tree was reconstructed using the nearest-neighbor-interchange (NNI) heuristic method, with partial deletion at an 80% site-coverage cutoff and five discrete gamma categories. Branch support was assessed using 1000 bootstrap replicates.

### 2.4. Preliminary Optimization of Extraction Conditions Using Ferulic Acid as a Representative Phenolic Marker

Leaf powder of *Ferula sinkiangensis* was used for preliminary optimization of the extraction conditions. Ferulic acid was selected as a representative phenolic marker to compare the effects of different extraction parameters rather than as a comprehensive measure of extraction efficiency for all metabolite classes. The ferulic acid reference standard was purchased from Shanghai Yuanye Bio-Technology Co., Ltd. (Shanghai, China). Ferulic acid was quantified at 320 nm using a UV-2700 UV–Vis spectrophotometer (Shimadzu Instruments (Suzhou) Co., Ltd., Suzhou, China) with an external calibration curve prepared from the reference standard, and the extraction yield was expressed as milligrams of ferulic acid per gram of dry leaf material. All experiments were performed in triplicate.

Single-factor experiments were initially conducted to determine suitable ranges for ethanol concentration, liquid-to-solid ratio, and extraction time. For each experiment, 0.5000 g of *F. sinkiangensis* leaf powder was accurately weighed, and one factor was varied while the remaining conditions were kept constant. To evaluate the effect of ethanol concentration, samples were extracted with 30%, 40%, 50%, 60%, or 70% ethanol (analytical grade, Tianjin Zhiyuan Chemical Reagent Co., Ltd., Tianjin, China) at a liquid-to-solid ratio of 10 mL/g for 30 min at an ultrasonic power of 300 W. The effect of the liquid-to-solid ratio was subsequently evaluated at 10, 20, 30, 40, and 50 mL/g using 50% ethanol for 30 min at 300 W. Finally, extraction times of 10, 20, 30, 40, and 50 min were compared using 50% ethanol at a liquid-to-solid ratio of 20 mL/g and an ultrasonic power of 300 W. Appropriate factor ranges for response surface optimization were selected based on the ferulic acid yields obtained from the single-factor experiments.

Based on the single-factor results, ethanol concentration (A), liquid-to-solid ratio (B), and extraction time (C) were selected as independent variables. A three-factor, three-level Box–Behnken design (BBD) [32] was employed for response surface analysis, with ferulic acid extraction yield used as the response variable. The coded levels of the three factors are presented in Table S3, and the experimental design and corresponding results are shown in Table S4. A quadratic polynomial model was fitted to evaluate the effects of the individual factors and their interactions on ferulic acid yield. The predicted extraction conditions were subsequently verified experimentally in triplicate.

### 2.5. Sample Preparation for Untargeted Metabolomic Profiling

Sample preparation for untargeted metabolomic profiling was performed with reference to established plant LC–MS metabolomics protocols, with modifications based on the preliminary extraction optimization conducted in this study [33,34]. Shade-dried Ferula leaves were ground into a fine powder and passed through an 80-mesh sieve. An accurately weighed portion (0.5000 g) of each sample was transferred to a capped centrifuge tube and extracted with 11 mL of 50% ethanol (analytical grade, Tianjin Zhiyuan Chemical Reagent Co., Ltd., Tianjin, China), corresponding to a liquid-to-solid ratio of 22 mL/g. Ultrasonic extraction was performed using an XM-P22H ultrasonic cleaner (Xiaomei Ultrasonic Instruments Co., Ltd., Kunshan, China) at 300 W for 35 min. These conditions were established on the basis of the preliminary extraction optimization using *F. sinkiangensis* and were subsequently applied consistently to all samples to standardize sample preparation for comparative metabolomic analysis.

### 2.6. UHPLC–MS/MS Conditions

Untargeted metabolomic profiling was performed using a Vanquish UHPLC system (Thermo Fisher Scientific, Waltham, MA, USA) coupled to a Q Exactive HF-X Orbitrap mass spectrometer (Thermo Fisher Scientific, Waltham, MA, USA). Chromatographic separation was carried out on an ACQUITY UPLC BEH C18 column (100 mm × 2.1 mm, 1.7 μm; Waters Corporation, Milford, MA, USA). Mobile phase A consisted of water containing 2% acetonitrile and 0.1% formic acid, whereas mobile phase B consisted of acetonitrile containing 0.1% formic acid. The flow rate was 0.3 mL/min, the injection volume was 1 μL, and the column temperature was maintained at 40 °C. The gradient elution program was as follows: 0–3.5 min, 2–50% B; 3.5–6.0 min, 50–95% B; 6.0–6.5 min, 95% B; 6.5–6.6 min, 95–2% B; and 6.6–8.0 min, 2% B. Mass spectrometric detection was performed using an electrospray ionization source in both positive- and negative-ion modes. The full-scan mass range was set to *m*/*z* 70–1050. The sheath and auxiliary gas flow rates were set to 50 and 13 arbitrary units, respectively. The heater and capillary temperatures were maintained at 425 °C and 325 °C, respectively. The spray voltage was set to 3.5 kV in positive-ion mode and −3.0 kV in negative-ion mode, and the ion funnel RF level was set to 50. Data-dependent MS/MS acquisition was performed using stepped normalized collision energies of 20%, 40%, and 60%. The resolution was set to 60,000 for full MS scans and 15,000 for MS/MS scans.

### 2.7. Data Processing and Statistical Analysis

Raw LC-MS data were processed using Progenesis QI v3.0 software, including baseline filtering, peak detection, peak integration, retention time correction, and peak alignment. A comprehensive data matrix containing retention time, *m*/*z*, peak intensity, and metabolite annotation information was subsequently generated. Metabolite annotation was performed based on accurate precursor masses, MS/MS fragmentation patterns, and database matching using Progenesis QI v3.0, with a mass tolerance of less than 10 ppm. Quality control (QC) samples were prepared by pooling equal aliquots of all sample extracts and were periodically injected throughout the analytical sequence to monitor instrument stability and data reproducibility. Total ion chromatograms (TICs) were used to evaluate chromatographic separation performance and signal stability during data acquisition. The normalized LC-MS peak intensity matrix was uploaded to MetaboAnalyst 6.0 for multivariate statistical analyses [35], including principal component analysis (PCA), partial least squares discriminant analysis (PLS-DA), hierarchical cluster analysis (HCA), and heatmap visualization, to explore metabolic variation and clustering patterns among *Ferula* samples. Variable importance in projection (VIP) scores derived from the PLS-DA model were used to assess the contribution of individual metabolites to sample discrimination. VIP-ranked metabolites were further visualized using heatmap analysis to compare their relative accumulation patterns across samples. Venn diagrams were constructed based on the occurrence of annotated metabolites among different *Ferula* groups to visualize shared and unique metabolites and to further characterize interspecific metabolic variation.

## 3. Results

### 3.1. Morphological Characteristics

The investigated *Ferula* species exhibited the general morphological characteristics of the genus, including erect stems, highly divided compound leaves with expanded petiole sheaths, compound umbels, yellow to yellowish flowers, and dorsiventrally compressed fruits (Figure 1). Considerable variation was observed among species in plant height, branching pattern, degree of leaf division, inflorescence architecture, and fruit morphology. However, the vegetative organs, particularly the leaves, showed substantial morphological similarity among several closely related species. Basal leaves were generally petiolate and repeatedly pinnately or ternately divided, whereas cauline leaves became progressively reduced toward the upper part of the stem. Reliable morphological identification of *Ferula* therefore depended largely on a combination of vegetative and reproductive characters, particularly inflorescence and fruit traits. These diagnostic reproductive characters are unavailable during the vegetative stage and may also be absent from harvested, processed, or commercially circulated leaf materials. Furthermore, variation associated with developmental stage and growing environment may increase intraspecific morphological variability and overlap among species. Consequently, although morphological characteristics provide an essential basis for taxonomic authentication of complete plant specimens, leaf morphology alone has limited discriminatory power for reliable identification of *Ferula* species. This morphological similarity supports the use of molecular and metabolomic approaches for further authentication and comparative characterization.

### 3.2. Phylogenetic Analysis

The NJ tree constructed from the concatenated ITS2, *trnH–psbA*, *rps11*, and *atpA–trnR* sequences clearly separated the investigated *Ferula* accessions from the three selected Apiaceae outgroups, *Angelica sinensis*, *Bupleurum commelynoideum*, and *Chamaesium jiulongense* (Figure 2). Based on the overall NJ topology, the *Ferula* accessions were provisionally divided into four broad phylogenetic groups for subsequent comparison. Bootstrap support varied considerably among internal nodes, indicating that several terminal relationships were better resolved than the deeper branching pattern.

Group I comprised *F. teterrima*, *F. sinkiangensis*, *F. conocaula*, *F. fukanensis*, and *F. lehmannii*. These taxa occupied a relatively coherent region of the NJ tree and were therefore treated as a broad garlic-odor *Ferula* assemblage in the subsequent chemotaxonomic comparison. Several terminal relationships within this group received moderate to relatively high support. For example, the two *F. lehmannii* accessions clustered together with 88% bootstrap support, whereas the broader relationships among *F. fukanensis*, *F. lehmannii*, *F. conocaula*, *F. sinkiangensis*, and *F. teterrima* were less strongly supported. Some *F. teterrima* and *F. sinkiangensis* accessions were interspersed among adjacent terminal branches, indicating incomplete resolution among these closely related taxa.

Group II contained *F. akitschkensis* and *F. syreitschikowii*. Group III included *F. ovina*, *F. dissecta*, *F. kirialovii*, *F. olivacea*, unidentified *Ferula* accessions, and *F. feruloides* accession 1. Group IV mainly comprised *F. songarica* together with *F. feruloides* accessions 2 and 3, whereas *F. feruloides* accession 1 was positioned separately in Group III. This incongruent placement of conspecific *F. feruloides* accessions further illustrates the limited resolution of some closely related samples in the NJ reconstruction.

For species represented by both wild and cultivated accessions, the NJ tree showed generally consistent grouping patterns across cultivation status. Wild and cultivated samples were largely interspersed within the same broad species-associated regions, with no distinct cultivation-related clustering observed. To further compare the NJ-based relationships, an ML analysis was performed using the same final taxon set (Figure S2). The ML tree recovered several terminal relationships also observed in the NJ tree, including the close association between *F. fukanensis* and *F. lehmannii*, several local relationships among *F. teterrima* and *F. sinkiangensis* accessions, and the association between *F. songarica* and some *F. feruloides* accessions. The NJ and ML trees showed agreement in several terminal and local relationships, whereas some differences were observed in deeper branching patterns. Overall, the concatenated four-locus barcode dataset separated the investigated *Ferula* accessions into broad genetic groups and revealed several consistent local relationships across the NJ and ML analyses.

### 3.3. Optimization of Extraction Conditions and Response Surface Analysis

An ultrasound-assisted extraction procedure was optimized to establish standardized and reproducible sample preparation conditions for subsequent metabolomic analysis. Ferulic acid was selected as a representative phenolic marker for comparing the effects of different extraction parameters rather than as a comprehensive indicator of the extraction efficiency of all metabolite classes. Single-factor experiments showed that ethanol concentration, liquid-to-solid ratio, and extraction time affected ferulic acid yield to different extents (Figure S1). Based on these results, ethanol concentration (A), liquid-to-solid ratio (B), and extraction time (C) were selected as independent variables for response surface optimization. The coded factor levels employed in the BBD are presented in Table S3 [32], while the experimental matrix and corresponding ferulic acid yields are summarized in Table S4. The quadratic polynomial regression model generated by response surface analysis was as follows:

Y = 7.75 − 0.4117A + 0.4159B − 0.5850C + 0.3892AB − 0.2225AC − 0.4225BC − 2.07A^2^ − 1.34B^2^ − 0.6371C^2^

Analysis of variance demonstrated that the regression model was highly significant (F = 27.39, *p* = 0.0001), whereas the lack-of-fit term was not significant (*p* = 0.2704) (Table S5), indicating that the model adequately described the experimental data. The coefficient of determination (R^2^) and adjusted coefficient of determination (adjusted R^2^) were 0.9724 and 0.9369, respectively, indicating good agreement between the experimental and predicted responses. The coefficient of variation (CV) was 5.79%, further supporting the precision and reliability of the model. Ethanol concentration (A), liquid-to-solid ratio (B), and extraction time (C) significantly affected the response (*p* < 0.05), with extraction time showing the greatest effect based on the corresponding F-values. The quadratic terms A^2^, B^2^, and C^2^ were also significant, indicating nonlinear effects of the three variables within the investigated ranges. Among the interaction terms, only the interaction between liquid-to-solid ratio and extraction time (BC) was significant (*p* = 0.0444). Overall, the relative influence of the three factors followed the order C > A > B.

The response surface and contour plots further illustrated the effects and interactions of the three variables on ferulic acid yield (Figure 3). In particular, the interaction between liquid-to-solid ratio and extraction time was evident from the relatively steep response surface and elliptical contour pattern, consistent with the significant BC interaction identified by ANOVA.

Numerical optimization predicted optimal conditions of 49.507% ethanol, a liquid-to-solid ratio of 22.309 mL/g, and an extraction time of 34.735 min, corresponding to a predicted ferulic acid yield of 7.962 mg/g and a desirability value of 0.940. For practical application, the conditions were adjusted to 50% ethanol, a liquid-to-solid ratio of 22 mL/g, and an extraction time of 35 min. Under these adjusted conditions, the predicted yield was 7.955 mg/g. Three independent validation experiments yielded 7.85, 7.92, and 7.97 mg/g, corresponding to a mean experimental yield of 7.91 ± 0.06 mg/g and an RSD of 0.76%. The relative deviation between the experimental and predicted values was 0.52%, demonstrating good agreement between model prediction and experimental validation. These standardized extraction conditions were subsequently applied uniformly to all *Ferula* leaf samples for comparative untargeted metabolomic profiling.

### 3.4. Total Ion Chromatograms and Overview of Putatively Annotated Metabolites

The chromatographic profiles of the *Ferula* leaf extracts were examined using total ion chromatograms (TICs) acquired in positive- and negative-ion modes (Figure 4). Numerous chromatographic signals were detected throughout the analytical run, with relatively dense signal regions observed at retention times of approximately 0.8–3.0 min and 5.0–6.5 min. Positive- and negative-ion modes exhibited distinct but complementary signal profiles, reflecting differences in the ionization characteristics of the detected metabolites and increasing the overall coverage of the chemical constituents in the *Ferula* leaf extracts.

Based on accurate precursor masses, MS/MS fragmentation patterns, and database matching, a total of 1972 metabolites were putatively annotated in the leaves of 11 *Ferula* species. Terpenoids represented the largest chemical class, comprising 797 metabolites and accounting for 40.42% of all putatively annotated metabolites. These included sesquiterpenoids, diterpenoids, monoterpenoids, and triterpenoids, among which sesquiterpenoids comprised 225 metabolites (11.41%). Flavonoids constituted the second-largest class, with 485 metabolites (24.59%), followed by phenolic acids and derivatives with 253 metabolites (12.83%) and coumarins and derivatives with 177 metabolites (8.98%). Several characteristic sesquiterpene coumarins, including farnesiferol A, colladonin, conferone, and feropolidin, were classified within the coumarin and derivative category in the present dataset. Organic acids and derivatives, lignans and derivatives, quinones, and other less abundant chemical classes accounted for the remaining metabolites (Table S6). Overall, the metabolite annotation results revealed a chemically diverse profile in the investigated *Ferula* leaves and provided the basis for subsequent multivariate statistical and chemotaxonomic analyses.

### 3.5. Principal Component Analysis and Partial Least Squares Discriminant Analysis

PCA and PLS-DA were performed using the normalized LC–MS dataset to evaluate overall metabolic variation among the 11 *Ferula* species. In the PCA model, PC1 and PC2 explained 24.6% and 19.7% of the total variance, respectively, accounting for a cumulative variance of 44.3%. As shown in the PCA score plot (Figure 5A), most samples belonging to the same species tended to cluster together despite differences in geographical origin or wild and cultivated status. Several species occupied relatively distinct regions of the score plot, whereas others showed partial overlap, indicating varying degrees of interspecific metabolic differentiation. PERMANOVA based on the PCA score space further supported significant overall separation among the investigated *Ferula* species (F = 126.75, R^2^ = 0.9829, *p* = 0.005; 199 permutations).

PLS-DA was subsequently performed as a supervised approach to further examine species-associated metabolic patterns (Figure 5B). The overall sample distribution was broadly consistent with that observed in the PCA, with several species showing clear separation while others remained relatively close or partially overlapped. Cross-validation indicated limited predictive performance of the PLS-DA model. The two-component model yielded the highest Q^2^ value, with R^2^ = 0.2658 and Q^2^ = 0.0965. Although the three-component model showed a slightly higher R^2^ value, its Q^2^ decreased, indicating no further improvement in predictive performance. Nevertheless, permutation testing based on the between-/within-group separation statistic showed that the observed group separation was significantly greater than that obtained after random permutation of the class labels (1000 permutations, empirical *p* < 0.001). Accordingly, the PLS-DA was used to complement the PCA results by further characterizing group differentiation and identifying metabolic features with relatively high contributions to the observed variation. Detailed model validation parameters are summarized in Table S7. Overall, the PCA and PLS-DA analyses consistently revealed taxon-associated metabolic variation among the investigated *Ferula* samples, with both distinct and partially overlapping metabolic profiles observed across species.

VIP scores derived from the PLS -DA model were used as an exploratory criterion to evaluate the relative contributions of individual metabolic features to group differentiation (Figure 6). Considering the modest predictive performance of the PLS-DA model, VIP rankings were used to prioritize candidate discriminant metabolites and were interpreted together with the corresponding statistical and abundance patterns. Among the highest-ranked features were metabolites putatively annotated as dihydroxyacidissiminol, kiwiionoside, 4-carvomenthenol, feropolidin, farnesiferol A, umbelliferone, colladonin, hesperetin, nerolidol, conferone, and (E)-1-propene-1-sulfenic acid. These metabolites represented several major chemical classes, including coumarins, terpenoids, flavonoids, and sulfur-containing compounds. The candidate metabolites were subsequently examined by heatmap analysis to compare their relative accumulation patterns across species.

### 3.6. Hierarchical Cluster Analysis of Ferula Species

To further examine the metabolic relationships among the investigated *Ferula* species, HCA was performed using the normalized LC–MS dataset. Samples belonging to the same species generally clustered together, indicating that within-species metabolic similarity was greater than the variation observed among many of the investigated species. Based on the overall dendrogram topology (Figure 7), the 11 *Ferula* species were broadly separated into three major metabolic groups.

Group A comprised *F. songarica*, *F. akitschkensis*, *F. syreitschikowii*, and *F. olivacea*. Within this group, the three samples of each species clustered closely together, and *F. akitschkensis* and *F. syreitschikowii* showed relatively similar overall metabolic profiles. *F. songarica* and *F. olivacea* were also positioned within the same major metabolic group, although they formed distinct species-level subclusters.

Group B consisted of *F. feruloides* and *F. ovina*. The three samples of each species formed compact subclusters, and the two species were clearly separated from the remaining taxa at the major clustering level. Notably, all *F. feruloides* samples clustered together based on their metabolic profiles, despite the inconsistent placement of one accession in the DNA barcode-based phylogenetic tree. This pattern indicates that metabolomic profiling provided complementary chemical information for evaluating relationships among these samples.

Group C comprised *F. conocaula*, *F. sinkiangensis*, *F. lehmannii*, *F. fukanensis*, and *F. teterrima*. These species formed a distinct major metabolic group, with samples from the same species generally clustering together despite differences in geographical origin and wild or cultivated status. *F. sinkiangensis*, *F. fukanensis*, *F. lehmannii*, and *F. teterrima*, which have documented culinary use, were all included in this group. *F. conocaula* was also positioned within the same major cluster. In addition, all *F. teterrima* samples grouped together in the metabolomic dendrogram, including the accession that showed an atypical placement in the DNA barcode-based phylogenetic analysis.

Collectively, HCA revealed structured metabolic variation among the investigated *Ferula* species, with clear within-species clustering and three broader metabolic groupings. The metabolomic clustering showed both concordant and divergent patterns relative to the DNA-based phylogenetic relationships, indicating that molecular phylogeny and metabolic phenotype provide complementary perspectives on the relationships among *Ferula* resources. These results provide a basis for subsequent analysis of the metabolites contributing to the observed chemotaxonomic differentiation.

### 3.7. Comparison Between Wild and Cultivated Accessions

For species represented by both wild and cultivated accessions, no consistent separation according to cultivation status was observed across the phylogenetic and metabolomic analyses. In the NJ tree, wild and cultivated accessions were generally interspersed within the same broad taxon-associated regions. A similar pattern was observed in the PCA, where conspecific wild and cultivated samples tended to remain relatively close at the broad grouping level. In the HCA, wild and cultivated accessions of the same species were generally retained within the same major metabolic clusters rather than forming distinct cultivation-based groups. These results suggest that no consistent cultivation-associated separation was evident within the present sampling framework. This might be attributed to the relatively short cultivation history of *Ferula* in Xinjiang, where many cultivated provenances are derived from wild sources. Consequently, cultivated *Ferula* plants still retain considerable wild germplasm characteristics, and the influence of the cultivation environment on its metabolites is not sufficiently pronounced. Therefore, its independent effect could not be determined from the current dataset.

### 3.8. Venn Diagram Analysis of Annotated Metabolites

Venn diagram analysis was performed within the HCA-derived groups to compare the occurrence of shared and uniquely detected metabolites among the investigated *Ferula* species (Figure 8). A metabolite was considered present in a species when it was detected in at least two of the three samples representing that species.

In Group C, comprising *F. sinkiangensis*, *F. teterrima*, *F. conocaula*, *F. lehmannii*, and *F. fukanensis*, 1190 putatively annotated metabolites were shared among all five species (Figure 8A). In addition, 73, 41, 21, 11, and 6 metabolites were uniquely detected in *F. teterrima*, *F. conocaula*, *F. sinkiangensis*, *F. fukanensis*, and *F. lehmannii*, respectively. These results showed that the five species shared a substantial proportion of their detected metabolite repertoire while retaining differences in metabolite occurrence.

In Group B, comprising *F. feruloides* and *F. ovina*, 1596 metabolites were commonly detected in both species (Figure 8B), whereas 58 and 204 metabolites were uniquely detected in *F. feruloides* and *F. ovina*, respectively. This pattern indicated extensive overlap in metabolite occurrence between the two species, together with species-associated differences.

In Group A, comprising *F. songarica*, *F. akitschkensis*, *F. syreitschikowii*, and *F. olivacea*, 1339 metabolites were shared among all four species (Figure 8C). In addition, 51, 9, 40, and 31 metabolites were uniquely detected in *F. songarica*, *F. akitschkensis*, *F. syreitschikowii*, and *F. olivacea*, respectively. Thus, species within this group exhibited a large common metabolite repertoire while retaining differences in metabolite occurrence.

The Venn diagram analysis revealed extensive sharing of annotated metabolites among species within each HCA-derived group, together with a smaller number of uniquely detected metabolites. These presence patterns complemented the HCA-based grouping and provided additional information on interspecific metabolic variation among the investigated *Ferula* species.

### 3.9. Heatmap Analysis

Heatmap analysis was performed using the top 50 VIP-ranked metabolic features to further examine their relative accumulation patterns among the investigated *Ferula* samples (Figure 9), with detailed annotation information provided in Table 2. The heatmap revealed marked variation in the relative abundances of these metabolites among species and showed distinct species- and group-associated accumulation patterns.

Samples of *F. conocaula*, *F. sinkiangensis*, *F. fukanensis*, *F. teterrima*, and *F. lehmannii* exhibited broadly similar accumulation patterns for a substantial proportion of the VIP-ranked metabolites. Several coumarin- and sesquiterpene-coumarin-related metabolites, including colladonin, farnesiferol A, feropolidin, umbelliferone, and conferone, showed relatively high abundances in multiple samples within this group. The terpenoid nerolidol and the sulfur-containing metabolite (E)-1-propene-1-sulfenic acid also displayed relatively high abundance patterns in several of these species. Although species-specific variation remained evident, the overall accumulation pattern of these samples was broadly consistent with their grouping in the HCA.

In contrast, *F. songarica*, *F. olivacea*, *F. syreitschikowii*, and *F. akitschkensis* showed comparatively higher relative abundances of several flavonoid-related metabolites, including quercetin, isoquercitrin, myricetin, gossypin, and quercetin-3-gentiobioside. These species exhibited an accumulation pattern distinct from that observed for *F. conocaula*, *F. sinkiangensis*, *F. fukanensis*, *F. teterrima*, and *F. lehmannii*. *F. feruloides* and *F. ovina* also displayed characteristic metabolite accumulation patterns and were separated from the other major groups in the heatmap.

Overall, the heatmap demonstrated that variation in the relative abundances of coumarins, terpenoids, flavonoids, phenolic-related compounds, and sulfur-containing metabolites contributed to the metabolic differentiation among the investigated *Ferula* species. The accumulation patterns of the VIP-ranked features were broadly consistent with the HCA-derived grouping and provided further information on the chemical characteristics underlying the observed chemotaxonomic differentiation.

## 4. Discussion

Accurate identification of *Ferula* species based solely on leaf morphology remains challenging because closely related taxa often share highly similar vegetative characteristics, whereas many diagnostically informative traits are associated with flowers and fruits. This problem is particularly relevant for young leaves harvested before the reproductive stage or for processed leaf materials in which reproductive characters are unavailable. In the present study, the combined ITS2, *trnH–psbA*, *rps11*, and *atpA–trnR* barcode dataset generally supported the morphological identification of the investigated materials and revealed the broad phylogenetic structure of the sampled *Ferula* resources. Notably, *F. sinkiangensis*, *F. fukanensis*, *F. lehmannii*, *F. teterrima*, and *F. conocaula* were positioned within the same broad phylogenetic group, indicating relatively close phylogenetic affinity among these taxa. Nevertheless, several internal nodes showed relatively low bootstrap support, and a small number of accessions did not cluster with the remaining samples assigned to the same species. The additional ML analysis recovered several terminal relationships also observed in the NJ tree, whereas deeper relationships were only partially congruent between the two approaches and remained weakly resolved. Similar limitations have been reported for conventional DNA barcodes when applied to closely related or recently diverged plant taxa, where low sequence divergence, gene-tree paraphyly, and hybridization can reduce species-level resolution [36]. In addition, incomplete lineage sorting may generate discordance among gene trees and contribute to weak or conflicting support for recently diverged lineages [37]. Recent plastome-based studies have demonstrated that genome-scale chloroplast information can provide additional resolution for phylogenetic reconstruction and species identification within *Ferula* [15]. Thus, the four-locus barcode combination used here appears useful for routine authentication and broad relationship assessment, whereas additional genomic evidence may be required to resolve ambiguous species-level relationships.

Untargeted metabolomics provided a complementary chemical perspective on the relationships among the investigated *Ferula* species. A total of 1972 metabolites were putatively annotated, with terpenoids, flavonoids, phenolic acids, and coumarins representing the predominant chemical classes. This composition is broadly consistent with previous phytochemical investigations of *Ferula*, in which sesquiterpenes, sesquiterpene coumarins, sulfur-containing compounds, flavonoids, and phenolic acids have been recognized as important specialized metabolites [5,8,12]. The occurrence of characteristic constituents such as farnesiferol A, colladonin, conferone, feropolidin, and umbelliferone further reflected the specialized chemical features of the genus. More importantly, the unsupervised PCA and HCA showed a clear tendency toward taxon-associated metabolic structure. PERMANOVA further supported significant overall separation among the investigated species in PCA score space. Most conspecific samples clustered relatively closely despite differences in geographical origin and cultivation status, suggesting that taxon-associated metabolic characteristics remained detectable within the present sampling framework. However, plant metabolomes are dynamic phenotypes influenced by genetic background as well as environmental and developmental factors [24]. The observed patterns should therefore be interpreted as taxon-associated metabolic tendencies rather than invariant species-specific chemical signatures.

HCA, Venn analysis, and heatmap visualization further revealed structured chemical variation among the investigated *Ferula* species. The three HCA-derived groups shared a substantial proportion of metabolites but differed in their relative accumulation patterns, indicating that chemotaxonomic differentiation was associated largely with quantitative variation in specialized metabolites rather than simply with the presence or absence of individual compounds. Group C showed relatively higher accumulation of several coumarin-, terpenoid-, and sulfur-related metabolites, whereas Group A showed comparatively higher abundances of several flavonoid-related compounds. These differences may reflect variation in specialized metabolic pathways among taxa. In particular, the predominance of terpenoids in the annotated dataset suggests that terpenoid metabolism is an important component of chemical diversification in *Ferula*, while variation in coumarin- and flavonoid-related metabolism may also contribute to chemotaxonomic differentiation. Overall, the metabolomic classification of these *Ferula* resources appears to reflect the combined variation in metabolite composition and relative abundance.

A notable finding of the present study was the partial correspondence between DNA-based phylogenetic relationships and metabolomic grouping. The clearest example was the correspondence between phylogenetic Group I and metabolic Group C, in which *F. sinkiangensis*, *F. fukanensis*, *F. lehmannii*, *F. teterrima*, and *F. conocaula* were grouped together in both analytical frameworks. This pattern suggests that relatively close evolutionary relationships may be accompanied by similarities in the overall metabolic phenotype of *Ferula* leaves. Such correspondence is biologically plausible because specialized metabolite biosynthesis depends on genetically encoded enzymes and regulatory pathways, and related taxa may retain partially conserved metabolic capacities. However, this correspondence was not universal, indicating that metabolic similarity cannot be explained by phylogenetic relatedness alone. Environmental conditions, developmental stage, and cultivation history may also contribute to the observed discrepancies. Therefore, these findings support a partial correspondence between phylogenetic relationships and metabolomic grouping among the investigated *Ferula* species.

Conspecific wild and cultivated accessions generally showed similar broad grouping patterns in the NJ tree, PCA, and HCA. These similarities may reflect the retention of genetic background and associated metabolic characteristics during cultivation. Cultivation status was confounded with geographic origin, collection year, and environmental conditions, so these observations are interpreted within the present sampling framework. Future studies incorporating balanced sampling of conspecific wild and cultivated materials under comparable environmental and developmental conditions would help clarify cultivation-associated metabolic variation and inform the cultivation and quality evaluation of *Ferula* resources.

The clustering pattern of species with documented culinary use was also noteworthy. *F. sinkiangensis*, *F. fukanensis*, *F. lehmannii*, and *F. teterrima*, which have traditionally been consumed as leaf vegetables in Xinjiang [13,14], occurred within the same broad phylogenetic group and the same HCA-derived metabolic group. Sulfur-containing metabolites are of particular interest because sulfur compounds have been associated with the characteristic onion- or garlic-like odor of several *Ferula* resources [6,38]. These chemical similarities may provide clues to the traditional utilization and sensory characteristics of edible *Ferula* leaves. However, the present data do not establish direct relationships between individual metabolites and culinary acceptance, flavor, biological activity, or edible value. Such relationships require targeted quantitative, functional, and sensory validation. These findings therefore provide a chemical basis for future authentication and quality evaluation of *Ferula* leaf resources.

The present study provides an exploratory comparison of metabolic variation among 11 *Ferula* species, with three samples per species. Most conspecific samples showed generally consistent multivariate patterns across the different collection backgrounds. Geographical origin, collection period, and cultivation status were confounded in the sampling design, and the findings are interpreted within this sampling framework. Expanded, geographically balanced sampling and appropriate biological replication will support more detailed characterization of intraspecific variation and environmental influences [39,40]. The overall PLS-DA distribution was broadly consistent with the PCA results. Cross-validation of the two-component model yielded R^2^ = 0.2658 and Q^2^ = 0.0965. Accordingly, PLS-DA was used for exploratory assessment of taxon-associated metabolic differentiation, with VIP rankings providing candidate discriminant features for subsequent validation. Metabolites were putatively annotated using accurate precursor mass, MS/MS fragmentation patterns, and database matching, providing a chemical framework for interspecific comparison. Confirmation with authentic standards and targeted quantification are the next steps toward establishing the identities and abundance profiles of candidate discriminant metabolites. Ferulic acid served as a representative phenolic response marker for optimizing extraction and establishing standardized sample preparation. The optimization results describe extraction performance with respect to this marker and provide a consistent preparation procedure for comparative metabolomics. Evaluation using additional chemical markers will extend the assessment of extraction performance across metabolite classes. Future studies integrating larger and more balanced sample sets, standardized sampling conditions, targeted metabolite validation, and higher-resolution genomic data will further clarify the genetic and environmental contributions to the observed metabolic patterns and support the authentication and quality evaluation of *Ferula* leaf resources.

The present study demonstrates that DNA barcoding and untargeted metabolomics provide complementary genetic and chemical information for the authentication and chemotaxonomic evaluation of morphologically similar *Ferula* leaves. In particular, the shared grouping of taxa in phylogenetic Group I and metabolic Group C supports a partial correspondence between phylogenetic relationships and metabolic patterns. The grouping differences observed among other taxa further highlight the value of integrating multiple levels of evidence when evaluating *Ferula* resources.

## 5. Conclusions

This study integrated multi-locus DNA barcoding with LC–MS-based untargeted metabolomics to evaluate species authentication, phylogenetic relationships, and chemical diversity in 33 leaf samples representing 11 *Ferula* species from Xinjiang, China. The combined ITS2, *trnH–psbA*, *rps11*, and *atpA–trnR* barcode dataset provided molecular support for the authentication of most investigated samples and revealed broad phylogenetic relationships among the *Ferula* taxa, although several closely related accessions showed limited resolution. Untargeted metabolomic analysis resulted in the putative annotation of 1972 metabolites, with terpenoids, flavonoids, phenolic acids, and coumarins representing the predominant chemical classes. Multivariate analyses further revealed taxon-associated metabolic patterns and chemotaxonomic differentiation among the investigated species. Notably, the metabolomic grouping showed partial correspondence with the DNA-based phylogenetic relationships, particularly between phylogenetic Group I and metabolic Group C, suggesting that relatively close phylogenetic relationships may be accompanied by similarities in overall metabolic profiles. At the same time, the incomplete correspondence between the two analytical frameworks indicates that phylogenetic relatedness and metabolic phenotype provide related but non-identical information. The observed patterns represent exploratory taxon-associated metabolic trends within the present sampling framework. Expanded sampling, systematic assessment of environmental and cultivation factors, and confirmation of metabolite annotations with authentic standards provide directions for further validation. Overall, integrating DNA barcoding with untargeted metabolomics provides complementary molecular and chemical information for the authentication, comparative characterization, and chemotaxonomic evaluation of morphologically similar *Ferula* leaves, establishing a foundation for future resource authentication and quality evaluation.

## Figures and Tables

**Figure 1:**
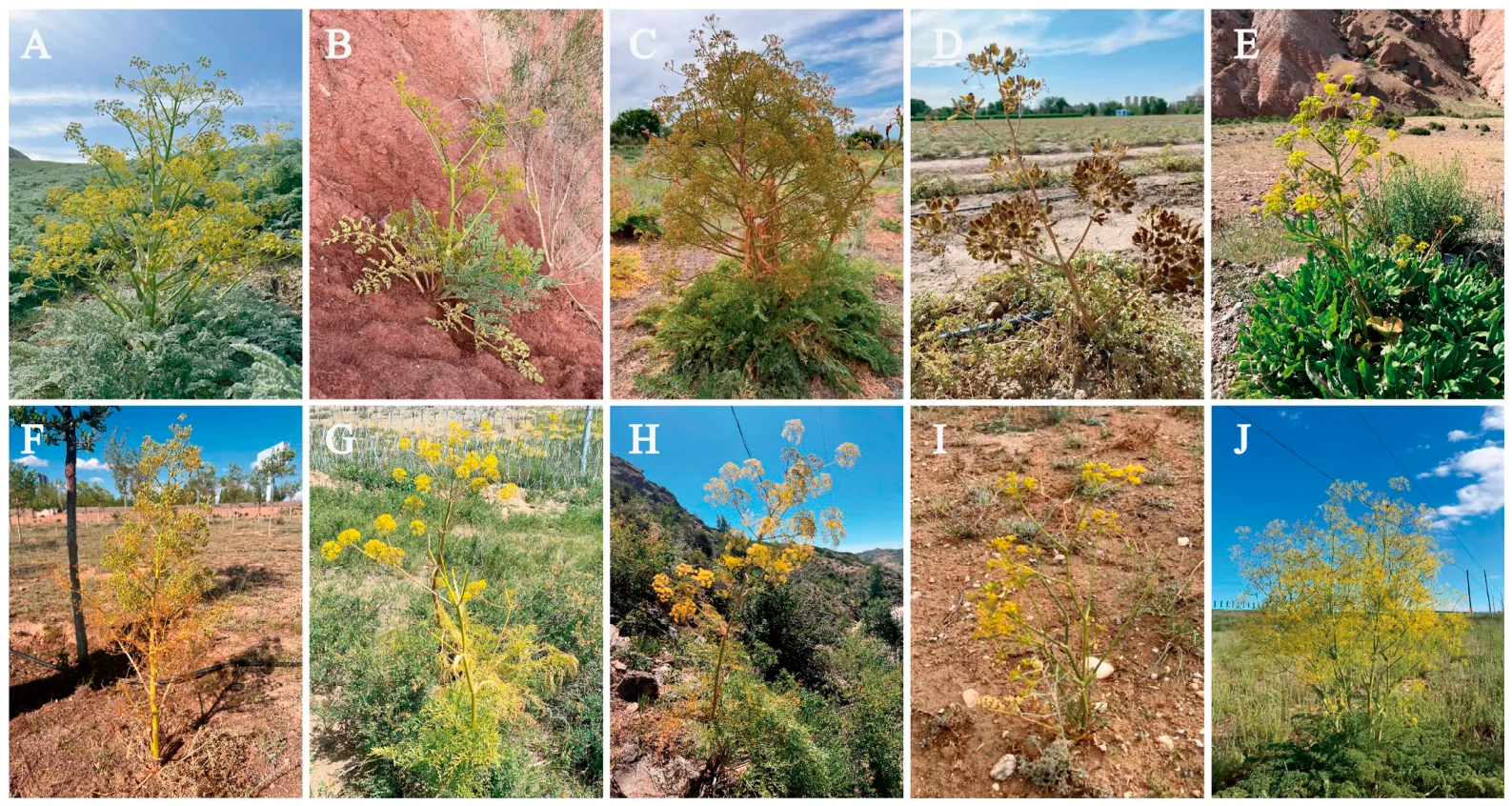
Morphological characteristics of representative *Ferula* species. (**A**) *F. sinkiangensis*, (**B**) *F. fukanensis*, (**C**) *F. teterrima*, (**D**) *F. lehmannii*, (**E**) *F. conocaula*, (**F**) *F. feruloides*, (**G**) *F. songarica*, (**H**) *F. syreitschikowii*, (**I**) *F. akitschkensis*, (**J**) *F. ovina*.

**Figure 2:**
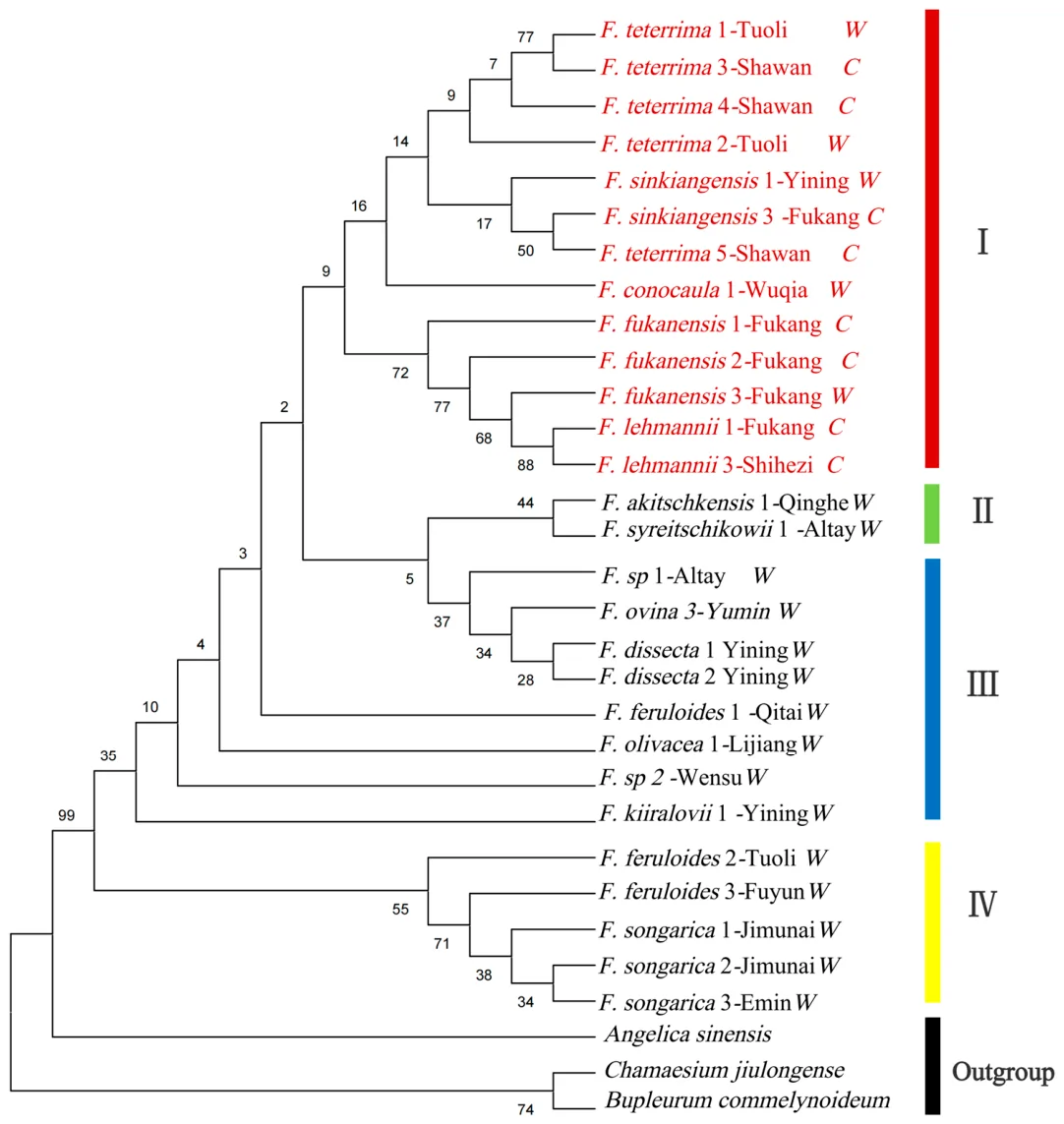
NJ phylogenetic tree of the investigated *Ferula* samples and three selected Apiaceae outgroups based on the concatenated ITS2, *trnH–psbA*, *rps11*, and *atpA–trnR* sequences. Colored bars indicate the four broad phylogenetic groups (I–IV) discussed in the text, and the black bar indicates the outgroup taxa. Numbers at the nodes represent bootstrap support values based on 1000 replicates. W, wild sample; C, cultivated sample.

**Figure 3:**
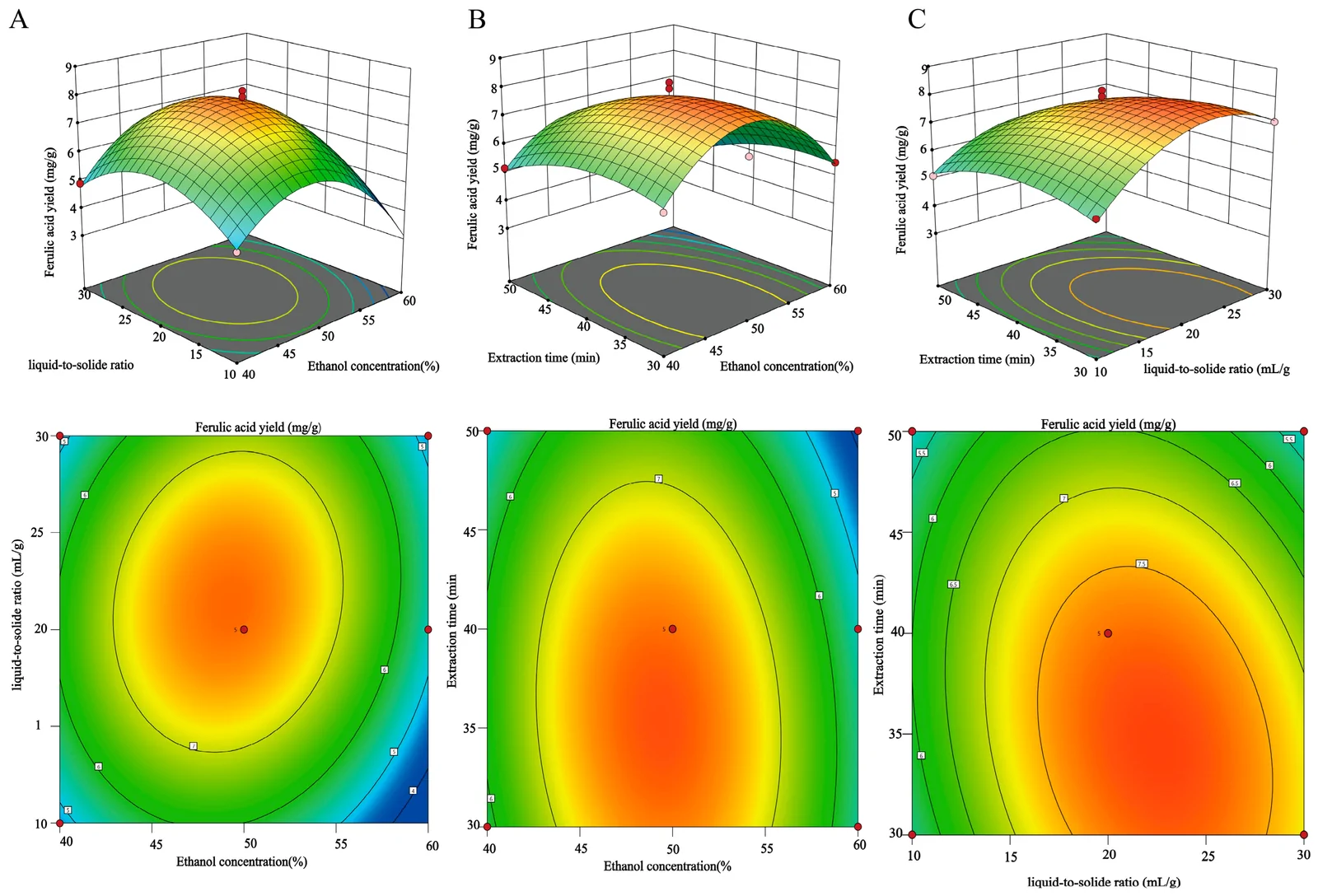
Response surface and contour plots illustrating the interactive effects of ethanol concentration (**A**), liquid-to-solid ratio (**B**), and extraction time (**C**) on ferulic acid yield.

**Figure 4:**
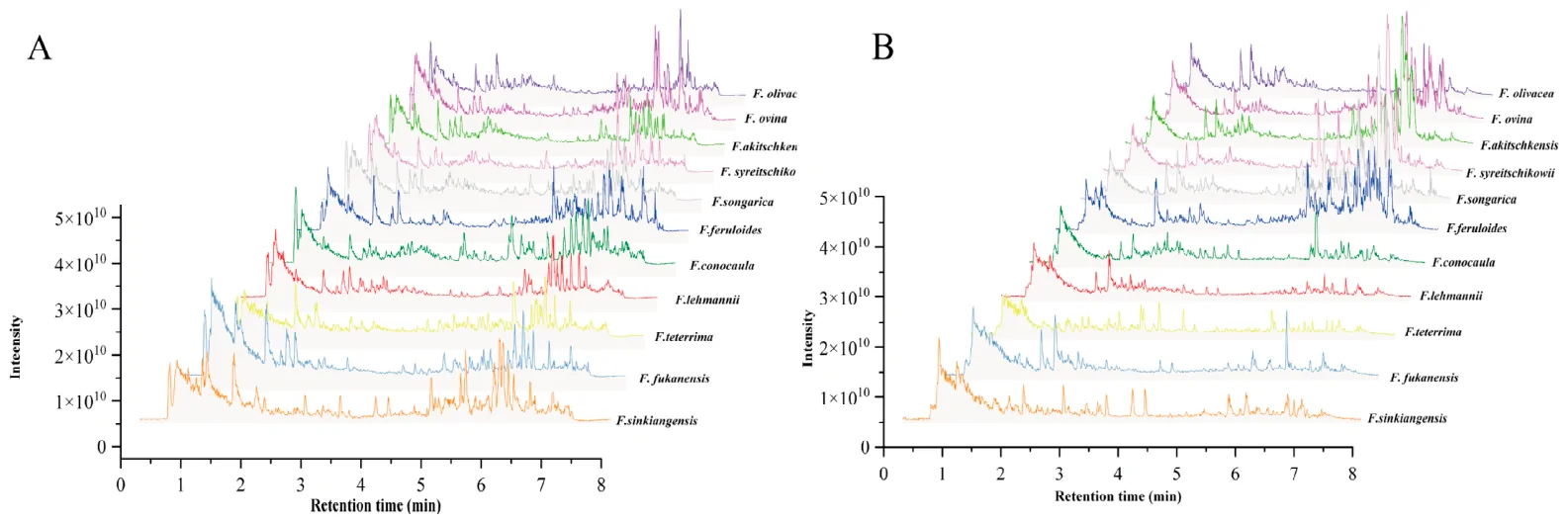
Representative TICs of leaf extracts from 11 *Ferula* species prepared under the optimized extraction conditions and analyzed by LC-MS. (**A**) ESI^+^ mode, (**B**) ESI^−^ mode.

**Figure 5:**
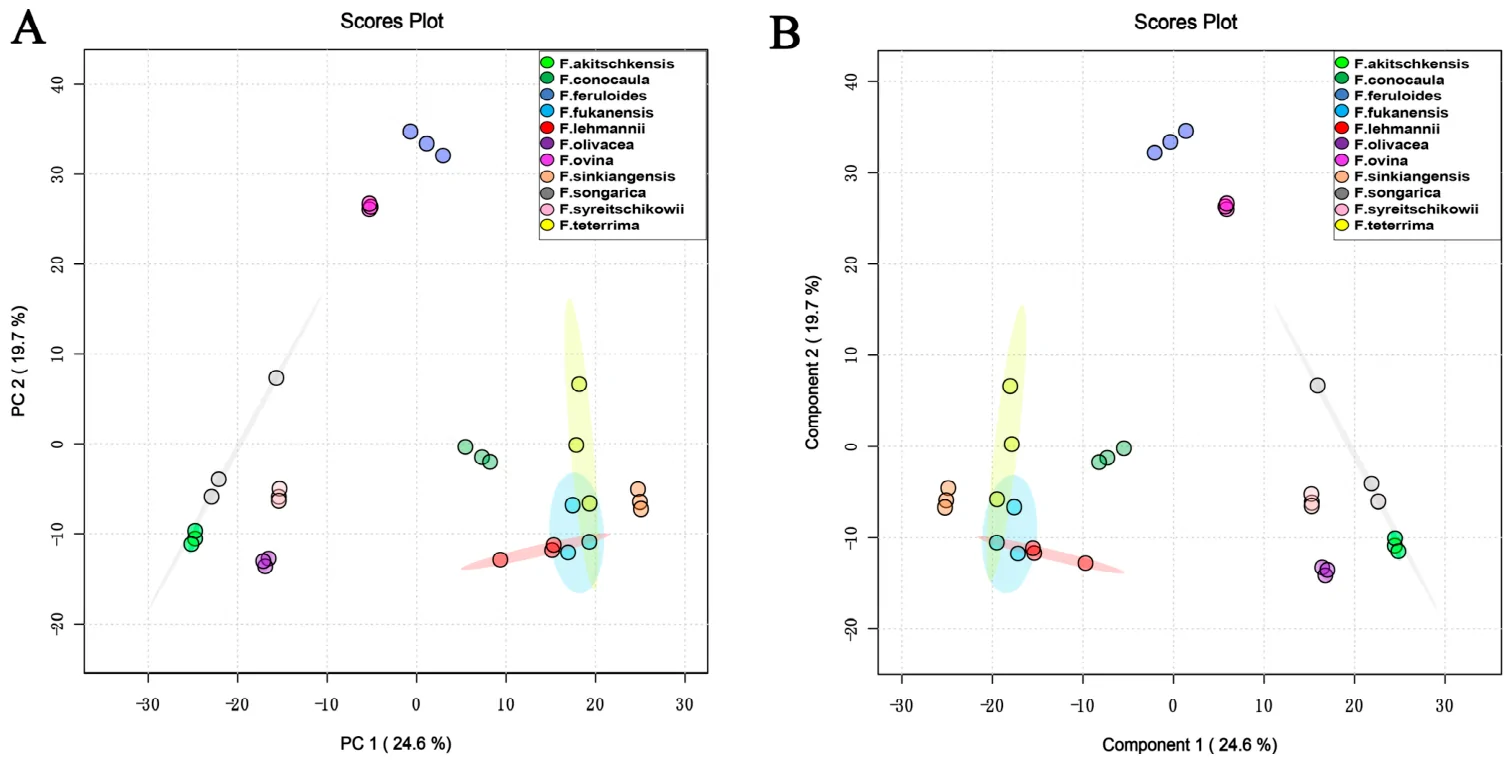
PCA and PLS-DA score plots based on the metabolomic profiles of leaves from 11 *Ferula* species. (**A**) PCA score plot; (**B**) PLS-DA score plot. Each point represents an individual sample, and different colors represent different *Ferula* species. The plots illustrate overall metabolic variation, within-taxon clustering, and separation among the investigated samples.

**Figure 6:**
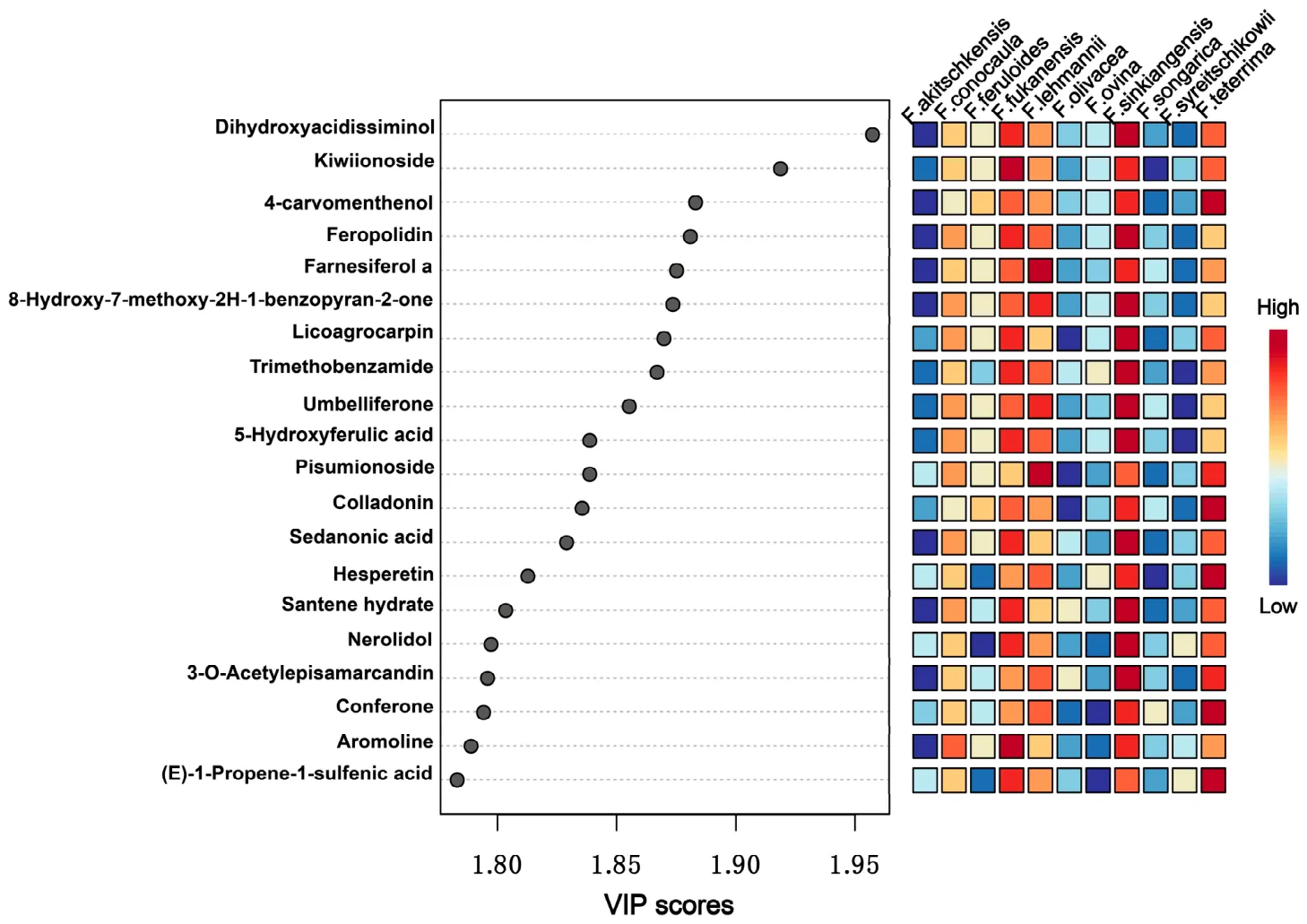
VIP scores and relative abundance patterns of metabolites contributing to separation among 11 *Ferula* species. VIP scores were derived from the PLS-DA model, with higher scores indicating greater contributions to the modeled group separation. The colored boxes show the relative abundance of the corresponding metabolites across the investigated species according to the color scale.

**Figure 7:**
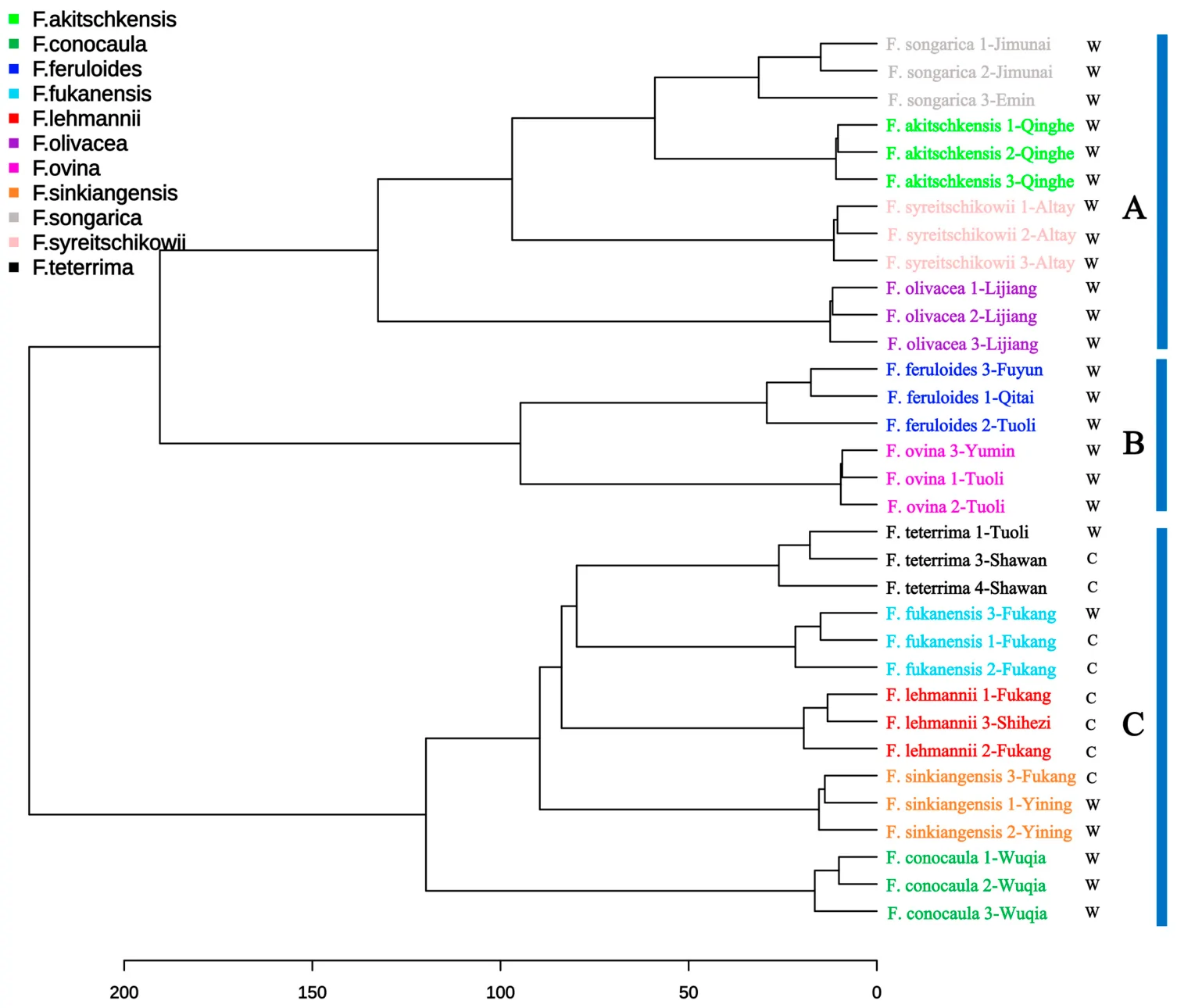
HCA of the metabolomic profiles of the investigated *Ferula* samples. Clustering was performed using Euclidean distance and Ward’s linkage. The scale at the bottom of the dendrogram represents clustering distance, with smaller values indicating greater similarity among samples. W, wild sample; C, cultivated sample.

**Figure 8:**
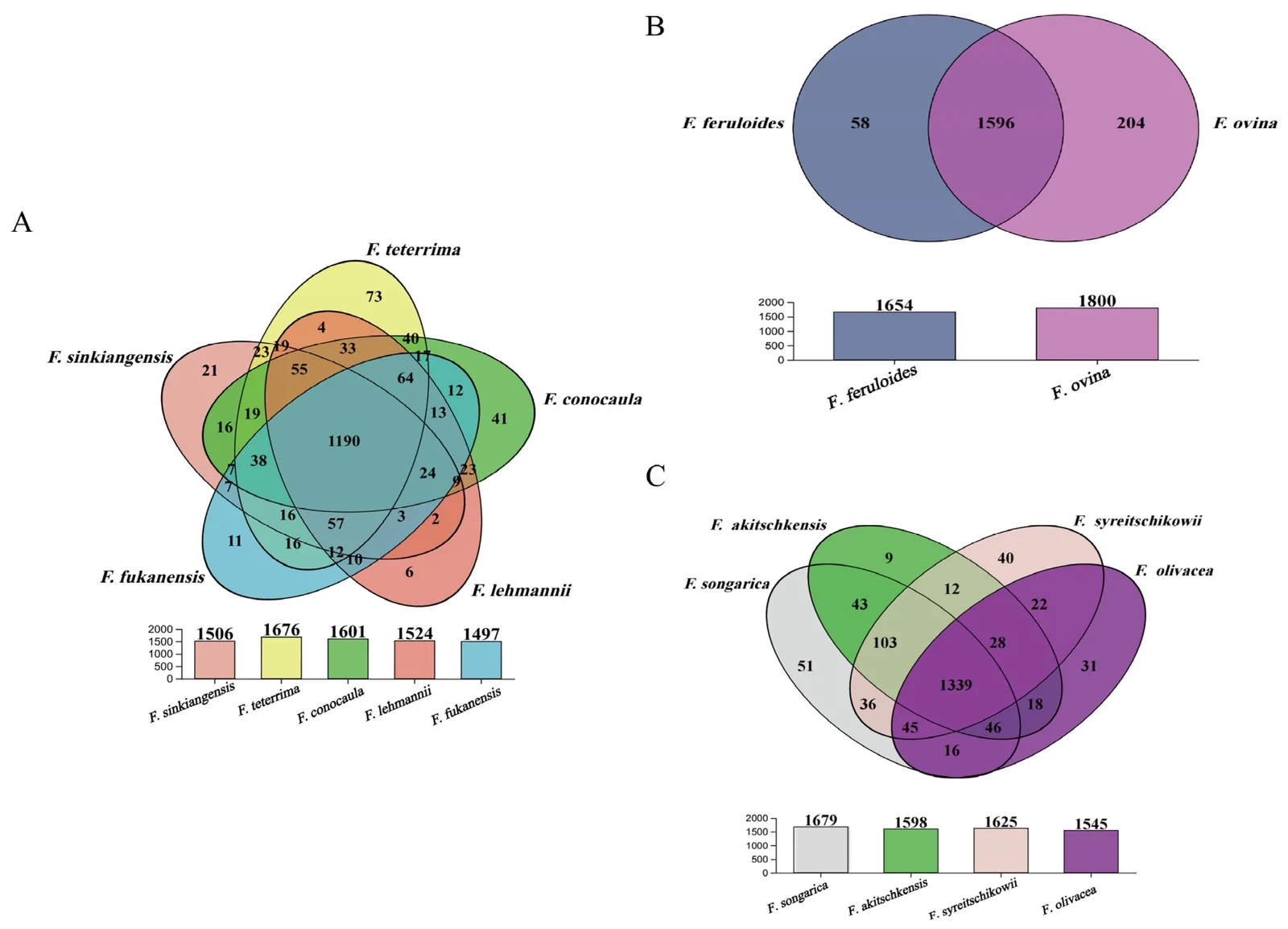
Venn diagram analysis of detected metabolites among HCA-derived *Ferula* groups. (**A**) Venn diagram of the group comprising *F. sinkiangensis*, *F. teterrima*, *F. conocaula*, *F. lehmannii*, and *F. fukanensis*. (**B**) Venn diagram of the group comprising *F. feruloides* and *F. ovina*. (**C**) Venn diagram of the group comprising *F. songarica*, *F. akitschkensis*, *F. syreitschikowii*, and *F. olivacea*. A metabolite was considered present in a given species when it was detected in at least two of the three samples or replicates assigned to that species.

**Figure 9:**
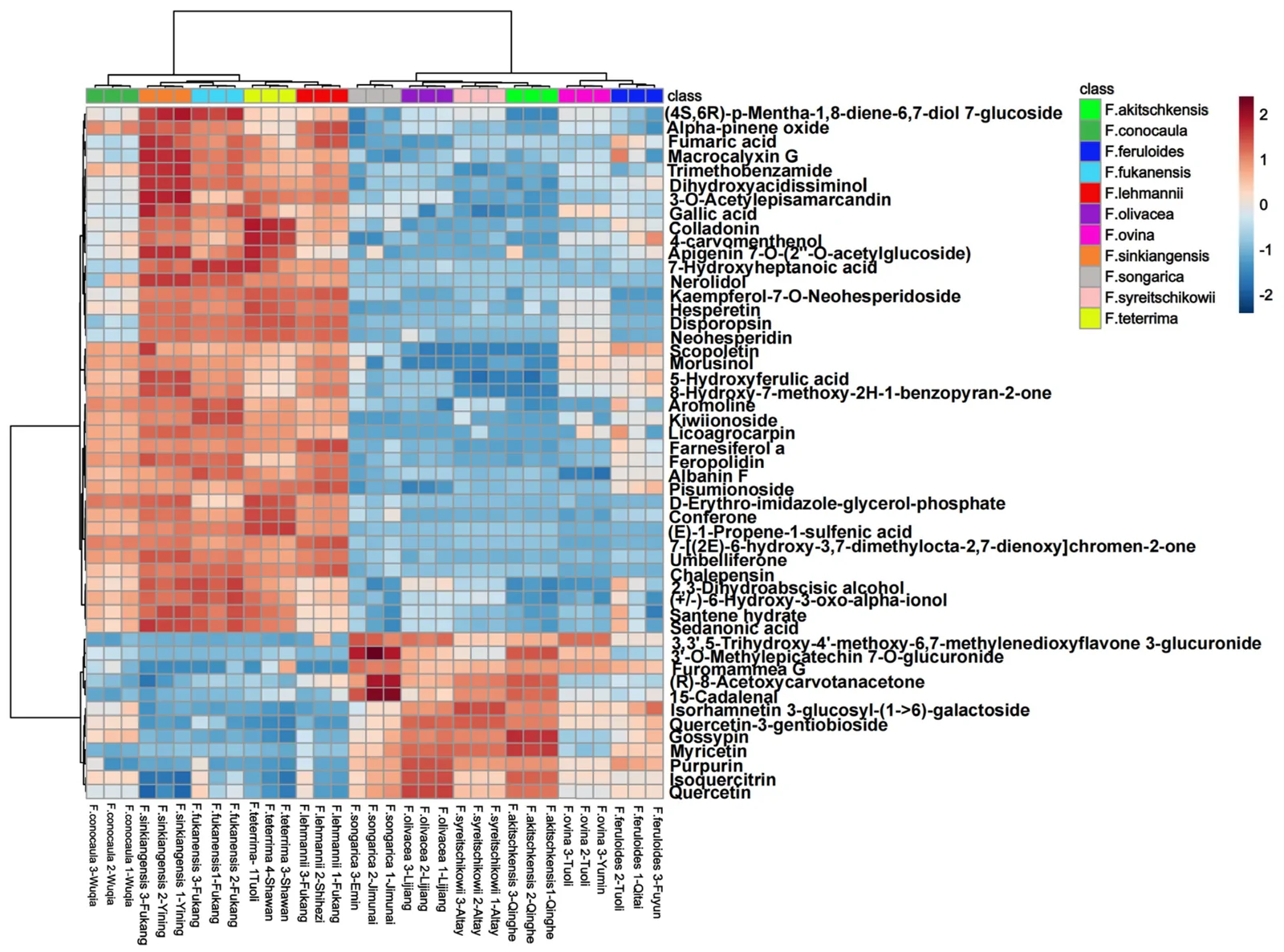
Heatmap analysis of the top 50 VIP-ranked metabolites from leaves of 11 *Ferula* species. Metabolites were selected according to VIP scores derived from the PLS-DA model. Rows represent metabolites, and columns represent individual samples. Red and blue indicate relatively high and low relative abundance, respectively. The color annotation above the heatmap represents different *Ferula* species. Samples belonging to the same species generally clustered together, and distinct metabolite accumulation patterns were observed among the investigated species. Detailed metabolite information is provided in Table 2.

**Table 1:** Primer information for amplification of DNA barcode regions in *Ferula* species.

Region	Primer Sequence (5′-3′)	Reference
ITS2	ITS2-F: ATGCGATACTTGGTGTGAAT ITS2-R: GACGCTTCTCCAGACTACAAT	[19]
*trnH-psbA*	trnH-F: CGTACCAAGTGATTTGTTTTACGAG psbA-R: ACCCAGTCCATCTGGAAATCTTGGTTC	[28]
*rps11*	rps11-F: TGGTCCTCTGCCGGTACTTGTG rps11-R: TCGGAGGTCTACAGCCATTATGTGG	[29]
*atpA-trnR*	atpA-F: AGGTTCAAATCCTATTGGACGCA trnR-R: TTTTGAAAGAAGCTATTCARGAAC	[30]

**Table 2:** Putatively annotated differential metabolites and their relative accumulation patterns in 11 *Ferula* species.

No.	RT (min)	Metabolites	Adduct	Measured *m*/*z*	Calculated *m*/*z*	Error (×10^−6^)	Formula	MS/MS Fragments	Class
1	1.90	(4S,6R)-p-Mentha-1,8-diene-6,7-diol 7-glucoside	**Adduct**	365.1355	365.1374	−5.3153	C_16_H_26_O_7_	203.0820 (100.00); 116.0495 (23.29); 101.0231 (19.07); 59.0127 (12.21); 74.0236 (9.05)	Terpenoids
2	5.44	Alpha-pinene oxide	[M+Cl]^−^	135.1169	135.1168	0.3838	C_10_H_16_O	107.0860 (100.00); 93.0707 (65.76); 135.1166 (63.66); 135.0426 (54.00); 91.0548 (46.63)	Terpenoids
3	0.96	Fumaric acid	[M+H−H_2_O]^+^	115.0025	115.0036	−9.8855	C_4_H_4_O_4_	73.0282 (100.00); 71.0127(97.82); 99.9248 (24.74); 114.9313 (15.66); 99.0076 (9.21)	Organic acids and derivatives
4	4.33	Macrocalyxin G	[M−H]^−^	425.2183	425.2181	0.5062	C_22_H_34_O_8_	115.0024 (100.00); 71.0126 (66.73); 425.2229 (64.30); 116.9274 (14.73); 99.9243 (8.40)	Terpenoids
5	4.23	Trimethobenzamide	[M−H]^−^	387.1922	387.1926	−0.9294	C_21_H_28_N_2_O_5_	387.1914 (100.00); 329.2329 (51.19); 211.1335 (21.36); 233.1152 (18.03); 341.1061 (13.78)	Phenolic acids and derivatives
6	5.83	Dihydroxyacidissiminol	[M−H]^−^	460.2695	460.2694	0.2429	C_25_H_33_NO_5_	383.2221 (100.00); 163.0390 (99.82); 203.1799 (57.65); 95.0863 (30.47); 133.1013 (26.68)	Phenolic acids and derivatives
7	5.53	3-*O*-Acetylepisamarcandin	[M+CH_3_OH+H]^+^	460.2694	460.2694	−0.0078	C_26_H_34_O_6_	203.1794 (100.00); 163.0390 (64.85); 383.2210 (53.68); 221.1901 (51.76); 119.0858 (36.85)	Coumarins and derivatives
8	1.24	Gallic acid	[M+NH_4_]^+^	169.0135	169.0143	−4.6531	C_7_H_6_O_5_	125.0232 (100.00); 169.0133 (44.72); 97.0285 (4.89); 69.0333 (4.37); 81.0332 (4.24)	Phenolic acids and derivatives
9	5.59	Colladonin	[M−H]^−^	383.2217	383.2217	−0.0683	C_24_H_30_O_4_	163.0391 (100.00); 383.2222 (71.26); 203.1798 (11.80); 147.1166 (9.66); 95.0862 (9.28)	Coumarins and derivatives
10	3.76	4-Carvomenthenol	[M+H]^+^	199.1334	199.1341	−3.4486	C_10_H_18_O	199.1337 (100.00); 155.1069 (17.87); 153.1284 (11.22); 57.0336 (9.11); 162.8382 (5.69)	Terpenoids
11	2.79	Apigenin 7-*O*-(2″-*O*-acetylglucoside)	[M+FA−H]^−^	473.1129	473.1089	8.4623	C_23_H_22_O_11_	191.0554 (100.00); 281.0486 (77.28); 473.1100 (29.76); 299.0598 (23.83); 59.0125 (14.71)	Flavonoids
12	3.13	7-Hydroxyheptanoic acid	[M−H]^−^	145.0860	145.0870	−6.7781	C_7_H_14_O_3_	99.0805 (100.00); 145.0860 (96.87); 100.9318 (19.18); 83.0490 (12.02); 115.9196 (4.75)	Organic acids and derivatives
13	6.15	Nerolidol	[M−H]^−^	223.2055	223.2056	−0.5005	C_15_H_26_O	81.0705 (100.00); 109.1016 (66.08); 123.1170 (58.08); 69.0706 (46.48); 95.0859 (45.18)	Terpenoids
14	2.66	Kaempferol-7-*O*-Neohesperidoside	[M+H]^+^	593.1518	593.1511	1.0994	C_27_H_30_O_15_	285.0403 (100.00); 593.1515 (51.76); 284.0324 (13.23); 133.0280 (3.14); 151.0030 (2.57)	Flavonoids
15	3.03	Hesperetin	[M−H]^−^	301.0723	301.0718	1.6290	C_16_H_14_O_6_	301.0716 (100.00); 164.0104 (30.39); 151.0027 (19.19); 136.0156 (11.67); 286.0476 (10.95)	Flavonoids
16	3.02	Disporopsin	[M+H]^+^	303.0862	303.0863	−0.3187	C_16_H_14_O_6_	153.0183 (100.00); 303.0867 (63.18); 177.0546 (54.29); 179.0338 (9.95); 117.0338 (8.10)	Flavonoids
17	3.03	Neohesperidin	[M+H]^+^	611.1971	611.1970	0.1178	C_28_H_34_O_15_	303.0860(100.00); 153.0184(49.95); 85.0290(46.77); 71.0498(37.69); 177.0546(31.30)	Flavonoids
18	6.80	Scopoletin	[M+H]^+^	193.0496	193.0495	0.4958	C_10_H_8_O_4_	193.0495 (100.00); 133.0285 (20.82); 178.0261 (13.12); 151.0387 (6.42); 137.0598 (5.92)	Coumarins and derivatives
19	5.47	Morusinol	[M+H]^+^	439.1755	439.1751	0.8360	C_25_H_26_O_7_	219.0649 (100.00); 151.0393 (64.21); 313.1435 (63.53); 245.0814 (59.94); 205.0493 (56.68)	Flavonoids
20	5.69	5-Hydroxyferulic acid	[M+H−H_2_O]^+^	193.0496	193.0496	0.2085	C_10_H_10_O_5_	193.0497 (100.00); 133.0288 (17.15); 178.0268 (11.19); 151.0390 (9.91); 137.0589 (5.13)	Phenolic acids and derivatives
21	5.17	8-Hydroxy-7-methoxy-2H-1-benzopyran-2-one	[M+H]^+^	193.0496	193.0496	0.1151	C_10_H_8_O_4_	193.0494 (100.00); 133.0284 (21.34); 178.0261 (13.26); 137.0597 (5.94);151.0392 (5.42)	Coumarins and derivatives
22	4.77	Aromoline	[M−H]^−^	593.2638	593.2657	−3.1491	C_36_H_38_N_2_O_6_	593.2649 (100.00); 80.9640 (48.69); 79.9564 (19.03); 225.0065 (14.58); 94.9795 (11.05)	Lignans and derivatives
23	3.78	Kiwiionoside	[M−H]^−^	405.2133	405.2130	0.6237	C_19_H_34_O_9_	405.2142 (100.00); 59.0127 (94.71); 73.0282 (64.37); 101.0218 (39.92); 363.2031 (33.12)	Terpenoids
24	3.45	Licoagrocarpin	[M+H]^+^	339.1574	339.1591	−4.9488	C_21_H_22_O_4_	339.1564(100.00); 177.0560(33.21); 241.0758(20.81); 145.0282 (19.23); 105.0701(13.28)	Flavonoids
25	6.00	Farnesiferol A	[M+H]^+^	383.2216	383.2217	−0.2155	C_24_H_30_O_4_	163.0391 (100.00); 203.1794 (30.29); 147.1169 (18.28); 95.0863 (15.46); 119.0857 (15.33)	Coumarins and derivatives
26	5.45	Feropolidin	[M+H]^+^	383.2216	383.2217	−0.2311	C_24_H_30_O_4_	163.0391 (100.00); 135.1168 (10.58); 383.2240 (9.60); 81.0706 (9.41); 107.0494 (7.88)	Coumarins and derivatives
27	1.85	Albanin F	[M+Cl]^−^	727.1941	727.1952	−1.5658	C_40_H_36_O_11_	87.0074 (100.00); 85.0283(63.43); 175.0235 (49.87); 59.0127 (47.11); 199.0238 (34.44)	Flavonoids
28	3.33	Pisumionoside	[M−H]^−^	403.1975	403.1973	0.4389	C_19_H_32_O_9_	403.1961 (100.00); 361.1867 (45.48); 59.0127 (36.77); 343.1758 (18.26); 71.0127 (16.52)	Terpenoids
29	3.25	D-Erythro-imidazole-glycerol-phosphate	[M−H]^−^	237.0259	237.0282	−9.7135	C_6_H_11_N_2_O_6_P	58.9949 (100.00); 195.0145 (9.31); 106.9983 (7.57); 177.0039 (5.63); 59.0125 (3.93)	Organic acids and derivatives
30	5.52	Conferone	[M+H−H_2_O]^+^	363.1953	363.1955	−0.4168	C_24_H_28_O_4_	201.1638 (100.00); 163.0391(93.14); 145.1014 (66.11); 159.1169 (65.19); 133.1014 (36.90)	Coumarins and derivatives
31	1.87	(E)-1-Propene-1-sulfenic acid	[M+H]^+^	91.0218	91.0212	6.3132	C_3_H_6_OS	73.0113 (100.00); 65.0393 (90.52); 62.9910 (41.03); 91.0215 (29.41); 56.9657 (8.48)	Organosulfur compounds
32	6.04	7-[(2E)-6-hydroxy-3,7-dimethylocta-2,7-dienoxy]chromen-2-one	[M+H−H_2_O]^+^	297.1484	297.1485	−0.4737	C_19_H_22_O_4_	297.1478 (100.00); 163.0390 (68.63); 93.0704 (17.59); 175.0384 (16.10); 107.0859 (15.80)	Coumarins and derivatives
33	6.18	Umbelliferone	[M+H]^+^	163.0389	163.0389	−0.2683	C_9_H_6_O_3_	163.0391 (100.00); 107.0496 (27.66); 119.0495 (15.04); 91.0548 (12.05); 135.0445 (1.79)	Coumarins and derivatives
34	6.35	Chalepensin	[M+H]^+^	255.1015	255.1016	−0.2940	C_16_H_14_O_3_	255.1010 (100.00); 175.0387 (46.44); 199.0390 (34.68); 163.0389 (22.44); 93.0704 (16.71)	Coumarins and derivatives
35	3.43	2,3-Dihydroabscisic alcohol	[M+FA−H]^−^	297.1707	297.1707	−0.0108	C_15_H_24_O_3_	297.1720 (100.00); 59.0127 (67.21); 83.0496 (41.93); 97.0650 (38.34); 143.0706 (33.78)	Terpenoids
36	5.72	(+/−)-6-Hydroxy-3-oxo-alpha-ionol	[M−H]^−^	223.1336	223.1340	−1.8036	C_13_H_20_O_3_	223.1331 (100.00); 195.1386 (20.63); 205.1226 (17.06); 179.1425 (8.42); 59.0160 (8.40)	Sesquiterpenoids
37	4.66	Santene hydrate	[M+FA−H]^−^	185.1177	185.1186	−4.7327	C_9_H_16_O	185.1175 (100.00); 141.1268 (34.79); 116.9275 (12.18); 85.0030 (4.01); 139.1121 (3.93)	Terpenoids
38	4.66	Sedanonic acid	[M−H]^−^	209.1179	209.1184	−2.1785	C_12_H_18_O_3_	209.1175 (100.00); 83.0492 (7.07); 163.1123 (5.51); 73.0108 (3.79); 165.1271 (3.75)	Organic acids and derivatives
39	3.01	3,3′,5-Trihydroxy-4′-methoxy-6,7-methylenedioxyflavone 3-glucuronide	[M−H]^−^	519.0784	519.0780	0.6829	C_23_H_20_O_14_	300.0272 (100.00); 475.0874 (54.58); 271.0248 (42.84); 59.0127 (23.36); 255.0298 (18.43)	Flavonoids
40	3.47	3’-*O*-Methylepicatechin 7-*O*-glucuronide	[M+H]^+^	481.1313	481.1340	−5.7106	C_22_H_24_O_12_	481.1242 (100.00); 437.1407 (20.11); 233.0786 (8.61); 271.0386 (3.22); 395.1260 (2.12)	Flavonoids
41	5.72	Furomammea G	[M+H]^+^	389.1958	389.1959	−0.2682	C_22_H_28_O_6_	95.0861 (100.00); 121.1015 (22.59); 151.0392 (19.92); 219.0651 (15.06); 85.0655 (12.00)	Coumarins and derivatives
42	6.03	(R)-8-Acetoxycarvotanacetone	[M+H−2H_2_O]^+^	175.1118	175.1118	0.1936	C_12_H_18_O_3_	133.1015 (100.00); 175.1119 (82.39); 119.0858 (54.45); 105.0702 (35.96); 147.1167 (16.44)	Terpenoids
43	5.51	15-Cadalenal	[M+H]^+^	213.1274	213.1274	0.1978	C_15_H_16_O	213.1277 (100.00); 195.1169 (34.16); 171.1163 (32.36); 157.1012 (21.76); 185.1329 (20.22)	Sesquiterpenoids
44	2.58	Isorhamnetin 3-glucosyl-(1->6)-galactoside	[M−H]^−^	639.1573	639.1567	0.9942	C_28_H_32_O_17_	314.0428 (100.00); 639.1541 (78.54); 299.0195 (67.69); 271.0244 (36.70); 243.0293 (24.87)	Flavonoids
45	2.44	Quercetin-3-gentiobioside	[M+H]^+^	627.1559	627.1556	0.5488	C_27_H_30_O_17_	303.0500 (100.00); 85.0291 (11.72); 61.0291 (6.06); 91.0393 (4.94); 97.0286 (4.56)	Flavonoids
46	2.47	Gossypin	[M−H]^−^	479.0834	479.0831	0.6041	C_21_H_20_O_13_	479.0823 (100.00); 317.0297 (47.26); 151.0028 (36.46); 299.0202 (31.44); 178.9977 (31.01)	Flavonoids
47	2.47	Myricetin	[M+H^]+^	319.0448	319.0448	−0.1079	C_15_H_10_O_8_	319.0445 (100.00); 153.0182 (18.17); 273.0390 (9.81); 245.0439 (7.94); 165.0183 (7.39)	Flavonoids
48	2.86	Purpurin	[M−H]^−^	255.0297	255.0299	−0.6362	C_14_H_8_O_5_	255.0295 (100.00); 227.0340 (8.24); 211.0391 (8.18); 183.0444 (4.19); 213.0185 (3.74)	Quinones
49	2.70	Isoquercitrin	[M+H]^+^	465.1029	465.1027	0.4192	C_21_H_20_O_12_	303.0498 (100.00); 85.0290 (5.57); 61.0291 (4.55); 229.0495 (4.33); 153.0182 (3.58)	Flavonoids
50	2.86	Quercetin	[M+H]^+^	303.0499	303.0500	−0.2297	C_15_H_10_O_7_	303.0498 (100.00); 153.0184(8.25); 229.0496 (8.22); 137.0233 (6.15); 257.0448 (4.35)	Flavonoids

**Note:** All listed metabolites were detected across the 11 *Ferula* species.

## Data Availability

The original contributions presented in this study are included in this Article/Supplementary Materials. Further inquiries can be directed to the corresponding author.

## Supplementary Materials

The following supporting information can be downloaded at https://www.mdpi.com/article/10.3390/metabo16100712/s1: Figure S1: Single-factor experiments for ferulic acid extraction yield. Figure S2: Maximum likelihood phylogenetic tree of the investigated *Ferula* accessions based on the concatenated four-locus barcode dataset. Table S1: Collection information and analytical applications of *Ferula* samples used in this study. Table S2: GenBank accession numbers of outgroup taxa used in phylogenetic analysis. Table S3: Independent variables and their coded levels employed in the response surface methodology optimization. Table S4: Box–Behnken experimental design and observed ferulic acid yields under different extraction conditions. Table S5: Analysis of variance (ANOVA) for the quadratic polynomial model of ferulic acid extraction yield. Table S6: Comprehensive list of 1972 metabolites putatively identified in the leaves of 11 *Ferula* species by LC–MS-based untargeted metabolomics. Table S7: PERMANOVA and PLS-DA validation results for the normalized LC–MS metabolomic dataset.
