# Supplementary material for: DNA Barcoding and Untargeted Metabolomics Reveal Phylogenetic and Chemotaxonomic Relationships Among 11 Ferula Species from Xinjiang, China
Source: Metabolites. 2026 Sep 24;16(10):712. doi: 10.3390/metabo16100712 (PMC13706696; doi:10.3390/metabo16100712)
Supplement: Supplementary file 1 [file metabolites-16-00712-s001.zip › Supplementary Materials.pdf]

Supplementary Materials

DNA Barcoding and Untargeted Metabolomics Reveal Phylogenetic and Chemotaxonomic Relationships Among 11 *Ferula* Species from Xinjiang, China

Shuak Halimubek [1], Pengyan Yan [1], Lei Chen [1,2], Jingjing Chen [1], Xiaoqing Zhang [1], Siyu Chen [1], Dongdong Wang [1,3,4,5], Haiyan Xu [1,3,4,5] and Xuejia Zhang [1,3,4,5,*]

[1] College of Traditional Chinese Medicine, Xinjiang Medical University, Urumqi 830017, China; xuah0529@163.com (S.H.); 17590823073@163.com (P.Y.); chenlei@bucm.edu.cn (L.C.); 17799160495@163.com (J.C.); 15699091569@163.com (X.Z.); atmzpl@163.com (S.C.); wang_tcm@126.com (D.W.); xhy66003@163.com (H.X.)
[2] School of Chinese Materia Medica, Beijing University of Chinese Medicine, Beijing 102401, China
[3] Institute of Traditional Chinese Medicine, Xinjiang Medical University, Urumqi 830017, China
[4] Xinjiang Laboratory of Famous Prescription and Science of Formulas, Xinjiang Medical University, Urumqi 830017, China
[5] Key Laboratory for Research on Planting Standards of Authentic Chinese Medicinal Materials in Xinjiang, Xinjiang Medical University, Urumqi 830017, China
* Correspondence: zhangxuejia@xjmu.edu.cn; Tel.: +86-0991-2110289

Table of contents

Figure S1: Single-factor experiments for ferulic acid extraction yield.

Figure S2: Maximum likelihood phylogenetic tree of the investigated *Ferula* accessions based on the concatenated four-locus barcode dataset.

Table S1: Collection information and analytical applications of *Ferula* samples used in this study.

Table S2: GenBank accession numbers of outgroup taxa used in phylogenetic analysis.

Table S3: Independent variables and their coded levels employed in the response surface methodology optimization.

Table S4: Box-Behnken experimental design and observed ferulic acid yields under different extraction conditions.

Table S5: Analysis of variance (ANOVA) for the quadratic polynomial model of ferulic acid extraction yield.

Table S6: Comprehensive list of 1,972 metabolites putatively identified in the leaves of 11 *Ferula* species by LC-MS-based untargeted metabolomics.（In a separate excel file）

Table S7： PERMANOVA and PLS-DA validation results for the normalized LC–MS metabolomic dataset.

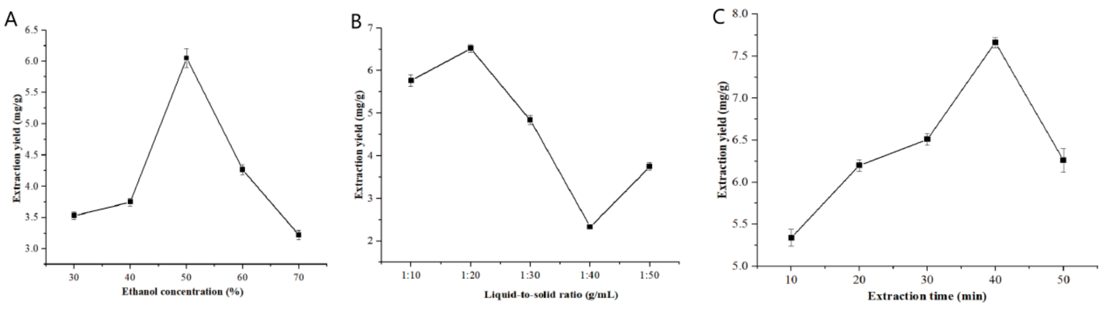

Figure S1. Single-factor experiments for ferulic acid extraction yield. A: ethanol concentration. B: liquid-to-solid ratio. C: extraction time affected.

Figure S2. Maximum Likelihood (ML) phylogenetic tree of the investigated *Ferula* accessions and three selected Apiaceae outgroups based on the concatenated ITS2, *trnH–psbA*, *rps11*, and *atpA–trnR* sequences. Numbers at the nodes indicate bootstrap support values based on 1,000 replicates. Taxa shown in red indicate the garlic-odor *Ferula* assemblage. W, wild sample; C, cultivated sample.

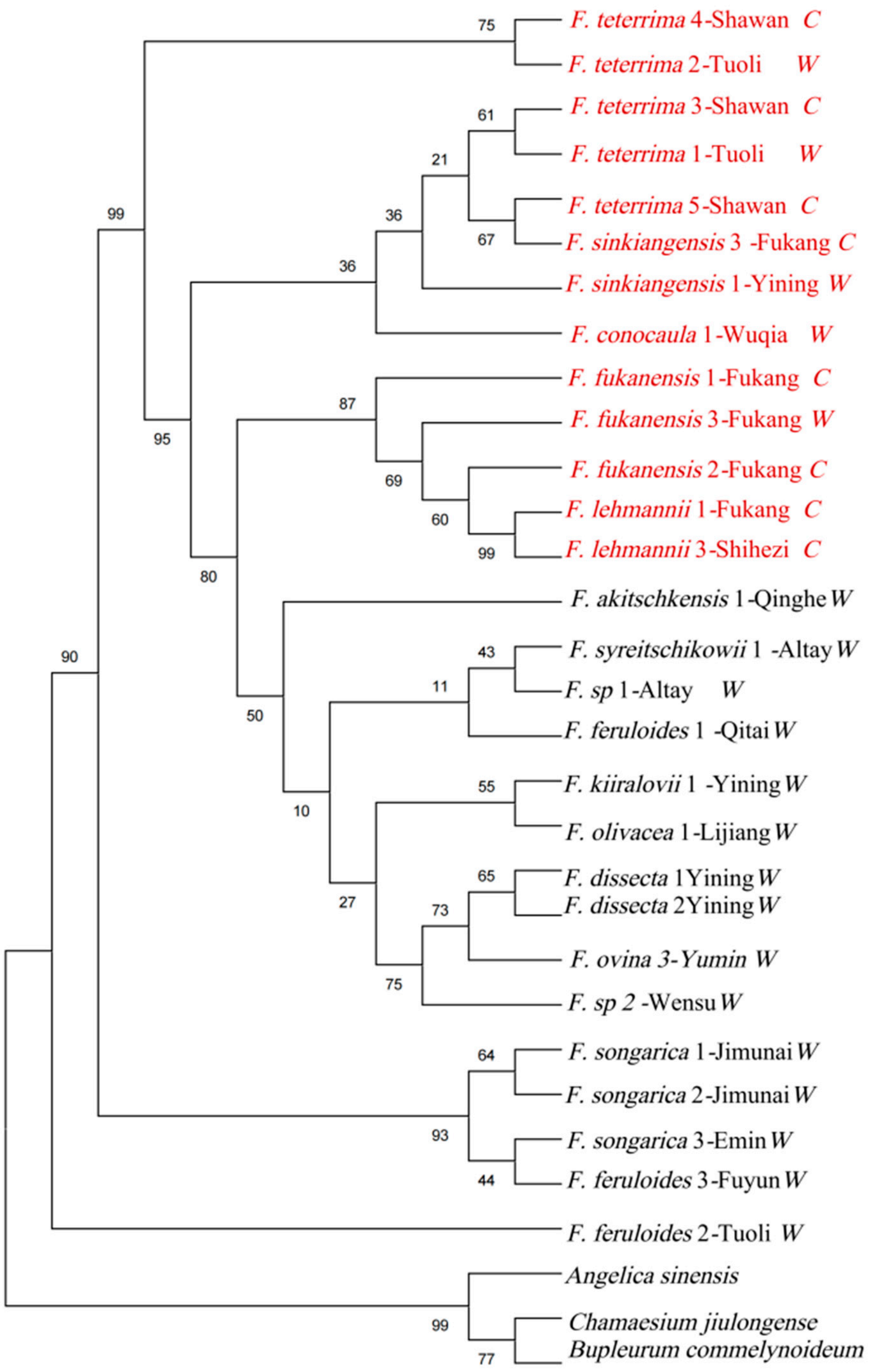

Table S1. Collection information and analytical applications of *Ferula* samples used in this study.

No.	Sample ID	Collection site	Collection time	Type	DNA barcoding	Untargeted metabolomics
1	*F. teterrima* 1-Tuoli	Tuoli	May 2023	W	Y	Y
2	*F. teterrima* 2-Tuoli	Tuoli	Jun 2023	W	Y	-
3	*F. Teterrima* 3-Shawan	Shawan	May 2025	C	Y	Y
4	*F. teterrima* 4-Shawan	Shawan	May 2025	C	Y	Y
5	*F. teterrima* 5-Shawan	Shawan	May 2025	C	Y	-
6	*F. sinkiangensis* 1-Yining	Yining	May 2023	W	Y	Y
7	*F. sinkiangensis* 2-Yining	Yining	May 2024	W	-	Y
8	*F. sinkiangensis* 3-Fukang	Fukang	May 2024	C	Y	Y
9	*F. conocaula* 1-Wuqia	Wuqia	Jul 2023	W	Y	Y
10	*F. conocaula* 2-Wuqia	Wuqia	Jul 2023	W	-	Y
11	*F. conocaula* 3-Wuqia	Wuqia	Jul 2023	W	-	Y
12	*F. fukanensis* 1-Fukang	Fukang	May 2024	C	Y	Y
13	*F. fukanensis* 2-Fukang	Fukang	May 2024	C	Y	Y
14	*F. fukanensis* 3-Fukang	Fukang	May 2023	W	Y	Y
15	*F. lehmannii* 1-Fukang	Fukang	May 2025	C	Y	Y
16	*F. lehmannii* 2-Fukang	Fukang	May 2025	C	Y	Y
17	*F. lehmannii* 3-Shihezi	Shihezi	May 2025	C	-	Y
18	*F. akitschkensis* 1-Qinghe	Qinghe	Aug 2023	W	Y	Y
19	*F. akitschkensis* 2-Qinghe	Qinghe	Aug 2023	W	-	Y
20	*F. akitschkensis* 3-Qinghe	Qinghe	Aug 2023	W	-	Y
21	*F. syreitschikowii* 1-Altay	Altay	Jul 2024	W	Y	Y
22	*F. syreitschikowii* 2-Altay	Altay	Jul 2024	W	-	Y
23	*F. syreitschikowii* 3-Altay	Altay	Jul 2024	W	-	Y
24	*F. sp.* 1-Altay	Altay	Jul 2024	W	Y	-
25	*F. ovina* 1-Tuoli	Tuoli	May 2023	W	Y	Y
26	*F. ovina* 2-Tuoli	Touli	May 2023	W	-	Y
27	*F. ovina* 3-Yumin	Yumin	May 2023	W	-	Y
28	*F. dissecta* 1-Yining	Yining	May 2024	W	Y	-
29	*F. dissecta* 2-Yining	Yining	May 2025	W	Y	-
30	*F. feruloides* 1-Qitai	Qitai	Jun 2024	W	Y	Y
31	*F. feruloides* 2-Tuoli	Tuoli	Jun 2024	W	Y	Y

32	*F. feruloides* 3-Fuyun	Fuyun	Jun 2024	W	Y	Y
33	*F. sp. 2*-Wensu	Wensu	Jul 2024	W	Y	-
34	*F. olivacea*1-Lijiang	Lijiang	Jul 2024	W	Y	Y
35	*F. olivacea* 2-Lijiang	Lijiang	Jul 2024	W	-	Y
36	*F. olivacea* 3-Lijiang	Lijiang	Jul 2024	W	-	Y
37	*F. kirialovii*-Yining	Yining	May 2025	W	Y	-
38	*F. songarica* 1-Jimunai	Jimunai	Apr 2024	W	Y	Y
39	*F. songarica* 2-Jimunai	Jimunai	Apr 2024	W	Y	Y
40	*F. songarica* 3-Emin	Emin	Apr 2024	W	Y	Y

Note: W, wild samples; C, cultivated samples. DNA barcoding indicates samples included in the phylogenetic analysis. Metabolomic profiling indicates samples used for LC–MS-based untargeted metabolomic analysis.

Table S2. GenBank accession numbers of outgroup taxa used in phylogenetic analysis.

Taxa	Voucher Source	Locality	ITS2	trnH–psbA	rps11	atpA–trnR
Bupleurum commelynoideum	GenBank	China	GU269874.1	HQ688204.1	OR778865.1	OR778865.1
Chamaesium jiulongense	JLJK6	Sichuan, China	PV810482.1	MN119368.1	MN119368.1	MN119368.1
Angelica sinensis	GenBank	China	LC773996	MK090027	NC_029393.1	NC_029393.1

Table S3. Independent variables and their coded levels employed in the response surface methodology optimization.

Symbols	Independent variables	Level		
		-1	0	+1
A	Ethanol concentration(%)	40	50	60
B	liquid-to-solide ratio(mL/g)	10	20	30
C	Extraction time(min)	30	40	50

Table S4. Box–Behnken experimental design and observed ferulic acid yields under different extraction conditions.

No	A (%)	B (mL/g)	C (min)	CUM (mg/g)
1	50	20	40	7.62
2	60	20	40	4.78
3	40	20	30	5.68
4	50	30	30	7.15
5	40	10	40	4.66
6	50	20	40	7.52
7	50	20	40	7.64
8	50	10	50	5.12
9	40	30	40	4.9
10	50	20	40	8.21
11	60	30	40	4.92
12	50	20	40	8.00
13	50	10	30	5.66
14	40	20	50	5.17
15	60	20	50	4.08
16	60	20	30	5.48
17	50	30	50	4.92

Table S5. Analysis of variance (ANOVA) for the quadratic polynomial model of ferulic acid extraction yield.

Source	Sum of Squares	DF	Mean Square	F-value	P-value	Significance
Model	29.45	9	3.27	27.39	0.0001	**
A	1.14	1	1.14	9.54	0.0176	*
B	0.9727	1	0.9727	8.14	0.0246	*
C	2.74	1	2.74	22.92	0.0020	**
AB	0.3285	1	0.3285	2.75	0.1412	
AC	0.1980	1	0.1980	1.66	0.2389	
BC	0.7140	1	0.7140	5.98	0.0444	
A^2	16.66	1	16.66	139.50	< 0.0001	**
B^2	5.98	1	5.98	50.03	0.0002	**
C^2	1.53	1	1.53	12.82	0.0090	*
Residual	0.8362	7	0.1195			
Lack of Fit	0.4917	3	0.1639	1.90	0.2704	
Pure Error	0.3445	4	0.0861			
Total	30.28	16				

Notes: A, ethanol concentration (%); B, liquid-to-solide ratio (mL/g); C, extraction time (min).

R=0.9861, R^2=0.9724, R^2adj=0.9369, C.V.%=5.79

$P < 0.05$ significant differences; $P < 0.01$ extremely significant differences

Table S7. Statistical validation results for PERMANOVA and PLS-DAanalyses.

Analysis	Model/Statistic	Result
PERMANOVA	F-value	126.75
PERMANOVA	R^2	0.9829
PERMANOVA	p-value	0.005
PERMANOVA	Permutations	199
PLS-DA cross-validation	Optimal components	2
PLS-DA cross-validation	R^2	0.2658
PLS-DA cross-validation	Q^2	0.0965
PLS-DA permutation test	Empirical p-value	< 0.001
PLS-DA permutation test	Permutations	1000

Notes: PERMANOVA, permutational multivariate analysis of variance; PLS-DA, partial least-squares discriminant analysis; R^2, goodness of fit; Q^2, predictive ability of the model.
